# Formation of an auditory sensory representation in posterior striatum emerges during a brief temporal window of associative learning in normal and hearing-impaired gerbils

**DOI:** 10.3389/fnsys.2025.1642595

**Published:** 2025-09-29

**Authors:** Jared B. Smith, Sean S. Hong, Damian J. Murphy, Shrivaishnavi Chandrasekar, Evelynne Dangcil, Jacqueline Nacipucha, Aaron Tucker, Nicolas L. Carayannopoulos, Sofia Carayannopoulos, Eran Peci, Matthew Y. Kiel, Nikhil Suresh, Maureen Guirguis, Umut A. Utku, Nihaad Paraouty, Jennifer D. Gay, P. Ashley Wackym, Justin D. Yao, Todd M. Mowery

**Affiliations:** ^1^Molecular Neurobiology Laboratory, Salk Institute for Biological Studies, San Diego, CA, United States; ^2^Department of Head and Neck Surgery and Communication Sciences, Rutgers Robert Wood Johnson Medical School, New Brunswick, NJ, United States; ^3^Rutgers Brain Health Institute, New Brunswick, NJ, United States

**Keywords:** posterior striatum, associative learning, auditory striatum, tail striatum, awake behaving recording, *in vivo* electrophysiology, *in vitro* whole cell recording

## Abstract

**Introduction:**

The posterior tail of the striatum receives dense inputs from sensory regions of cortex and thalamus, as well as midbrain dopaminergic innervation, providing a neural substrate for associative sensory learning. Previously, we have demonstrated that developmental hearing loss is associated with aberrant physiological states in striatal medium spiny neurons (MSNs).

**Methods:**

Here we directly investigated auditory associative learning impairments in the striatum of adult Mongolian gerbils that underwent transient developmental hearing loss or sham hearing loss during the critical period of auditory development. We used electrophysiology to reveal significant changes to neuronal population responses *in vivo* and intrinsic and synaptic properties to medium spiny neurons *in vitro* as animals learned an appetitive “Go/No-Go” auditory discrimination task. For *in vivo* experiments a 64-channel electrode was implanted in the auditory region of the posterior tail of the striatum and neuronal recordings were carried out as animals learned the task. For *in vitro* experiments, corticostriatal slice preparations were made from animals on each day of training.

**Results:**

In naïve animals from both groups there was limited to no phase locking to either auditory stimulus *in vivo*, and long term depression resulted from theta burst stimulation *in vitro*. Furthermore, intrinsic and synaptic properties in normal hearing animals were unaffected; however, the hearing loss group continued to show lowered synaptic inhibition, synaptic hyperexcitation, and suppressed intrinsic excitability in the hearing loss group. Starting around day 3–4 in both groups, the emergence of striatal medium spiny neuron phase locking to the auditory conditioning stimuli was observed *in vivo*. This occurred contemporaneous to an increased probability of theta burst induced LTP during MSN whole cell recording *in vitro*, and acquisition of the task as the correct rejection response significantly increased in the behaving animals. During the acquisition phase MSNs in the normal hearing group showed a significant decrease in synaptic inhibition and increase in synaptic excitation with no change to intrinsic excitability, while the MSNs in the hearing loss group showed a significant increase in synaptic inhibition, reduction of synaptic hyper excitability, and compensatory changes to intrinsic excitability that supported normal action potential generation. In both groups, synaptic properties were resolved to similar level of E/I balance that could be part of a conserved learning state.

**Discussion:**

These changes to the intrinsic and synaptic properties likely support LTP induction *in vivo* and the strengthening of synapses between auditory inputs and MSNs that facilitate neuronal phase locking. These findings have significant implications for our understanding of striatal resilience to sensory impairments in early life, in addition to establishing a granular understanding of the striatal circuit changes that support reward driven stimulus–response learning.

## Introduction

The basal ganglia is a series of subcortical structures that form extensive recurrent loops with the neocortex, to govern behavioral learning and dopamine-dependent extra-pyramidal motor output in all animals (Groenewegen, 2003). Among the connected structures of the basal ganglia, the striatum is essential for associative reward learning, decision making, and habit formation (Arber and Costa, 2022; Cox and Witten, 2019; Redgrave et al., 2010). Many decades of research have shown that the striatum receives extensive glutamatergic innervation from cortex, thalamus, and amygdala (Arber and Costa, 2022; Doig et al., 2010; Huerta-Ocampo et al., 2014). In mammals, the density of these inputs creates areas of limbic, cognitive, sensory and motor compartmentalization, as well as, regions of cross modal convergence throughout the striatum (Aoki et al., 2019; Hooks et al., 2018; Hintiryan et al., 2016; Voorn et al., 2004; Pan et al., 2010; Smith et al., 2019, 2022, 2025; Mowery et al., 2011, 2017; Alloway et al., 2006, 2009; Foster et al., 2021; Hoffer et al., 2005; Hoffer and Alloway, 2001; Paraouty and Mowery, 2021; Pidoux et al., 2011; Wright et al., 1999; Ponvert and Jaramillo, 2019; Reig and Silberberg, 2014, 2016; Shepherd, 2013; Zingg et al., 2014). Extensive striatal research has focused on the somatosensory and motor regions of the dorsal striatum (including dorsolateral regions of both caudate and putamen) (Balleine et al., 2007). These regions were thought to be essential for sensory motor associative learning involving action selection, goal-oriented behaviors, and habit formation (Barnes et al., 2005; Costa et al., 2004; Jog et al., 1999; Kubota et al., 2009; Yin, 2010).

Early studies in rodents, non-human primates, and cats suggested that there were auditory responsive regions in the posterior striatum (Apicella et al., 1991a, 1991b; Winer, 2005; Arnauld et al., 1996; LeDoux et al., 1991; Bordi and LeDoux, 1992; Hikosaka et al., 1989; McGeorge and Faull, 1989; Zhong et al., 2014), which receives overlapping innervation from somatosensory, motor, auditory and visual cortex (Reig and Silberberg, 2014). Posterior striatum is a key hub for associative sensory learning owing to the integration of sensory information about the stimuli from numerous thalamic nuclei, several primary and higher order sensory cortical regions, and basal lateral amygdala (Smith et al., 2019). Auditory-stimulus evoked responses in the tail of the striatum are thus a mixture of inputs from auditory thalamus and auditory cortex, allowing integration of different stimulus features extracted from parallel processing streams of information ascending the neuraxis, as has been described for visual (e.g., “what” vs. “where: pathway; Kravitz et al., 2013; Felleman and Van Essen, 1991) and somatosensory systems (Alloway, 2008; Chen et al., 2019; Guo et al., 2018, 2019; Nardoci et al., 2022; Ponvert and Jaramillo, 2019; Li et al., 2021). Furthermore, auditory cortical glutamatergic inputs have been shown to drive associative decision making in posterior striatum in mice (Znamenskiy and Zador, 2013). Subsequent research has verified that the tail of the striatum is a key structure for auditory based learning and behavior (Chen et al., 2022; Cui et al., 2025; Druart et al., 2025; Li et al., 2024; Ogata et al., 2022; Zhong et al., 2017). In fact, the excitatory drive from layer 5 corticostriatal neurons onto striatal medium spiny striatal neurons (MSNs) provides a logical driver for the neural plasticity that induces long term potentiation (LTP) and subsequently behavioral task acquisition through learning (Ghosh and Zador, 2021; Xiong et al., 2015).

The corticostriatal circuit has long been implicated in the etiology of many major diseases and disorders (Shepherd, 2013). Dysfunction along these circuits can lead to behavioral disorders, learning impairments, and psychiatric manifestations. We have previously shown that transient developmental hearing loss induces permanent changes in the intrinsic firing properties of striatal MSNs, as well as their excitatory and inhibitory synaptic function (Mowery et al., 2017). These changes were hypothesized to be maladaptive with the idea that they would likely lead to learning delays; however, instead we found that cellular membrane physiology and inhibitory synapse impairments were compensated for during a brief window of plasticity that allowed for normal auditory learning (Paraouty and Mowery, 2021). This suggested that striatal synaptic plasticity could drive associative conditioning in both normal and disordered circuits. To further investigate this, we carried out *in vivo* electrophysiological recordings and *in vitro* whole cell recordings from MSNs in the auditory region of the posterior tail of the Gerbil striatum, defined by the densest inputs from auditory cortex (Smith et al., 2025), while animals learned an auditory Go/No-Go discrimination task with repeated training over weeks. We found that a key feature of task acquisition is the emergence of the acoustic stimulus representation in the neuronal population response of striatal MSNs. The *in vitro* results confirmed that a brief window of synaptic plasticity involving changes to excitation and inhibition and cellular intrinsic excitability occur approximate to increased expression of theta burst induced LTP. Together, the learning induced changes could support synaptic modifications, such as LTP, that facilitate stimulus response encoding and strengthen MSN phase locking to the auditory stimuli.

## Experimental methods

### Animals

A total of (131) adult male (67) and female (64) Mongolian gerbils (*Meriones unguiculatus*) were used in this study. All animals were housed in the same vivarium facility under a 12/12 dark cycle with ad libitum access to food and water. Twenty-four male (12) and female (12) animals were used for the auditory discrimination learning task (NH Males = 6, NH Females = 6, HL Males = 6, HL Females = 6). Six normal hearing male (*N* = 3) and female (*N* = 3) animals and six hearing loss male (*N* = 3) and female (*N* = 3) animals were implanted with 64 channel electrode arrays in the posterior tail of the striatum and trained on the auditory Go/No-Go discrimination task. Twenty-five normal hearing male (*N* = 12) and female (*N* = 13) animals, and twenty-five hearing loss male (*N* = 13) and female (*N* = 12) animals were used for *in vitro* whole cell recordings of inhibitory post synaptic potentials (IPSPs). Eighteen normal hearing male (*N* = 9) and female (*N* = 9) animals and eighteen hearing loss male (*N* = 9) and female (*N* = 9) animals were used for *in vitro* whole cell recordings of excitatory post synaptic potentials and theta burst stimulation of MSNs across the corticostriatal circuit. Three male (*N* = 1) and female (*N* = 2) animals were used for neuroanatomical tracing of auditory cortex layer 5 inputs to the posterior tail of the striatum. All animal procedures were performed in accordance with the regulations of the Institutional Animal Care and Use Committee.

### Neuroanatomical tracing

Gerbils were anesthetized (isoflurane 2%) and placed in a stereotaxic frame. ACx stereotaxic locations were derived from coronal plane coordinates in the Mongolian gerbil atlas (Radtke-Schuller et al., 2016). The left temporal bone was exposed and a craniotomy was made at the level of core ACx (−2.35 bregma, ~4.5 mm ventral from the edge of the temporal bone), and the pipette was lowered orthogonally from the pial surface (DV 800 μm). AAV (AAV1-CaMKII-eGFP, 7 × 1,012 vg/mL) was injected with a Nanoject III (Drummond) at 10 nL per second until 350 nL was reached. The pipette was left in place for 20 min before being slowly withdrawn. At the end of the experiments, all injected animals were deeply anesthetized with an intraperitoneal injection of Euthasol (300 mg/kg) and perfused with phosphate-buffered saline and 4% paraformaldehyde. Brains were removed, postfixed, and sectioned at 50 μm on a benchtop vibratome (Pelco). All sections (50 μm) were float mounted on pig gel slides (Southern Biotech) and coverslipped with mounting medium (Invitrogen ProLong Antifade with DAPI). All images were generated on a Revolve 4 fluorescent imaging system.

### Developmental hearing loss

Reversible hearing loss was induced by inserting a malleable plug (BlueStik Adhesive Putty, RPM International Inc.) into the opening of each ear canal starting at P11. Animals were checked daily, and earplugs were adjusted to accommodate growth. Earplugs were removed at P23. Earplugs attenuate auditory brainstem responses and perceptual thresholds by approximately 35 dB, depending on frequency, and the attenuation is completely reversible (Mowery et al., 2015; Caras and Sanes, 2015).

### Auditory discrimination paradigm

Auditory decision-making was assessed in adult gerbils with an appetitive Go/No-Go associative conditioning paradigm. Adult gerbils were placed on controlled food access and trained to discriminate amplitude-modulated (AM) broadband noise (100% modulation depth) presented at 4 Hz (Go) versus 12 Hz (No-Go). Gerbil auditory decision-making task performance was conducted in a behavioral arena test cage (Med Associates) housed inside a sound-attenuating cubicle (Med Associates) or a sound attenuation booth (Whisper Room). Gerbils self-initiated trials by placing their nose in a nose port for a minimum of 100 ms that interrupted an infrared beam and triggered an acoustic stimulus. Each AM stimulus was initially presented at a sound pressure level (SPL) of 75 dB under normal-hearing conditions and had a 100 ms onset ramp to an unmodulated period of 500 ms that transitioned to an AM signal which lasted for 1.5 s. During “Go” trials (4 Hz), animals could approach a food trough on the opposite side of the cage and received a reward (20 mg dustless pellet; Bio-Serv). During “NoGo” trials (12 Hz), animals had to remove their snout and wait at least 600 ms before repoking to initiate the next trial. Go trials were scored as a Hit (correctly approaching the food trough) or Miss (failing to approach the food trough and repoking). NoGo trials were scored as a correct reject (CR; correctly repoking), or false alarm (FA; incorrectly approaching the food trough). After an FA trial, the house lights were turned off and another trial could not be initiated (time-out) for approximately 5 s. Behavioral choices were required within 10 s of trial onset for all trials. All task sessions are observed via a closed-circuit monitor. Stimuli, food reward delivery, and behavioral data acquisition were controlled by an iPac computer system running iCon behavioral interfaces (Tucker-Davis Technologies). Auditory stimuli were presented from a calibrated multifield speaker (MF1, Tucker-Davis Technologies) positioned 12 cm above the center of the cage. Sound calibration measurements were verified with a digital sound level meter prior to daily testing (Larson Davis SoundExpert 821 ENV).

### *In vivo* electrophysiological recordings

After behavioral shaping gerbils were implanted with a silicon probe with 64 recording sites (Neuronexus, model A4x16-5 mm-50-500-703-H64LP_30mm). The electrode array (4 shank, 16 channels/shank) was implanted in the left posterior tail of the striatum (posterior shank, bregma −2.15, 4.8 mm mediolateral, 3.0 mm dorsoventral) and extracellular single and multi-unit activity was recorded while animals learned the task. The probe was attached to a manual microdrive (Neuronexus, dDrive-XL) that allowed the electrode to be advanced and retracted across depth. Probes were inserted orthogonal to the pial surface. The surgical implantation procedure was performed under isoflurane anesthesia. Animals recovered for at least 1 week before being placed on controlled food access for further training. At the termination of each experiment, animals were deeply anesthetized with sodium pentobarbital (150 mg/kg) and perfused with phosphate-buffered saline and 4% paraformaldehyde. Brains were extracted, postfixed, and sectioned on a vibratome (Leica). Brightfield images were inspected under an upright microscope (Revolve Echo) and compared with a gerbil brain atlas (Radtke-Schuller et al., 2016) to verify the targeted striatal region.

Physiological data were acquired from tethered freely moving animals with a TDT digital head stage. Analog signals were preamplified and digitized at a 24.414-kHz sampling rate (PZ5, Tucker-Davis Technologies) and fed via fiber optic link to the RZ5 base station (Tucker-Davis Technologies) and then a PC running synapse (TDT) for online analysis, storage, and postprocessing. Offline, electrophysiological signals were isolated, and cluster sorted into single units with Offline Sorter software (Plexon). PCA sorting and the K-means method were used to isolate putative medium spiny neurons. Manual inspection of spike waveforms was conducted, aberrant spikes were removed, and well-isolated single units that displayed clear separation in principal component space, looked like medium spiny waveforms, and had spike widths greater than 1.0 ms were kept. All single units had distinct cluster cut waveforms and all interspike intervals were greater than 1 millisecond (indicating no multi-units were present in the waveform). Offline sorter putative single unit data was then exported to Neuroexplorer 5 (Plexon) for further analysis. A limited number of putative fast spiking units were recorded; however, there were not enough for meaningful statistical interpretation.

### Neural analyses

Timestamps were collected in Synapse and exported from Offline sorter into Neuroexplorer 5 (Plexon). For each Go and NoGo trial a time stamp (1.0 ms resolution) for the nose poke and trough entry allowed temporal PSTH reconstruction of the neural response to nose poke (−0.05 to 0 s), sound onset (non-modulated, 0 to 500 ms), modulated sound onset (0.5 to 1.5 s) and trough entry on Go hit and No-Go false alarm trials (−0.5 to 1.0 s). For training day 1 to 10, all units were normalized as neural probability PSTHs. To begin, all data was displayed in a PSTH for the Go Hit Trials (NH = 4,298 putative single units, HL = 4,451 putative single units). Data was curated so that only sound responsive neural traces remained with positive neural probability to the right of the nose poke between 0 and 0.5 s above 95% CI (NH = 3,529 putative single units, HL = 3,629 putative single units). Sound responsive units were largely recorded from shank two, three, and four which were the most posterior, with few units found on the first shank. This corresponds to the compartmentalized inputs from the auditory cortex (posterior tail) versus the somatosensory and motor inputs (anterior tail) in the Mongolian gerbil (Smith et al., 2025). All sound responsive neural data (NH = 2,147 putative single units, HL = 2,156 putative single units) was batch analyzed in neuroexplorer and exported to excel for analysis. In excel, data was divided into trial day (T1 to T10). Data was subdivided by (1) nose poke/stimulus data (−0.5 to 2.5 s) for Go Hit, No-Go FA, and No-Go CR and (2) trough data (−0.5 to 1.0 s) for Go Hit and No-Go FA. Trial days for behavioral epochs were based on each animals’ daily performance (*d*-prime) and divided into naïve (*d*-prime <1.5, T1–T2), acquisition (first 2 days *d*-prime >1.5, typically T3–T4 and sometimes T5), and mastery (*d*-prime >2.5, T9–T10). All data was divided in this fashion on an animal-by-animal basis and group analyzed. For the naïve behavioral epoch there were 649 putative single units for the NH group and 674 putative single units for the HL group. For the acquisition behavioral epoch there were 773 putative single units for the NH group and 745 putative single units for the HL group. For the mastery behavioral epoch there were 725 putative single units for the NH group and 737 putative single units for the HL group.

### Corticostriatal brain slice preparation

Brain slices were obtained within 3 h after a training/testing session. Animals were deeply anesthetized (isoflurane 3.0%) and perfused with ice cold artificial cerebrospinal fluid (ACSF, in mM: 125 NaCl, 4 KCl, 1.2 KH_2_PO_4_, 1.3 MgSO4, 26 NaHCO_3_, 15 glucose, 2.4 CaCl_2_, and 0.4 L-ascorbic acid; and bubbled with 95%O_2_-5%CO_2_ to a pH = 7.4). Brains were dissected into 4 °C oxygenated ACSF and a 25° cut was made through the right hemisphere. Each brain was vibratome-sectioned through the left hemisphere to obtain 300–400 mm peri-horizontal auditory corticostriatal slices. To validate the thalamo-recipient ACx, a bipolar stimulating electrode (FHC) was placed at the rostral border of the medial geniculate nucleus (MG). MG-evoked field responses were recorded in the ACx. To validate auditory cortico-recipient striatum, a bipolar stimulating electrode was placed in layer 5 ACx and ACx-evoked field responses were recorded in the striatum. Whole-cell current clamp recordings were obtained (Warner PC-501A) from striatal MSNs at 32 °C in oxygenated ACSF. Recording electrodes were fabricated from borosilicate glass (1.5 mm OD; Sutter P-97). The internal recording solution contained (in mM): 5 KCl, 127.5 K-gluconate, 10 HEPES, 2 MgCl2, 0.6 EGTA, 2 ATP, 0.3 GTP, and 5 phosphocreatine (pH 7.2 with KOH). The resistance of patch electrodes filled with an internal solution was between 5 and 10 MΩ. Access resistance was 15–30 MΩ and was compensated by about 70%.

### Cannula implantation and infusion

Gerbils were anesthetized (isoflurane 3%), placed in a stereotaxic frame, and the parietal, occipital, and frontal bones were exposed. The skin and sinew overlying these bones was removed from the surface of the skull. Two anchor screws were placed over the frontal cortex and secured in place with dental acrylic (Hereaus). Two craniotomies were made for bilateral cannula insertion into posterior tail of the striatum (Bregma −2.15, 4.8 mm mediolateral). Cannulae (Plastics One) were lowered to a depth of 3 mm from the skull surface and secured in place with dental acrylic (Hereaus). Dummy guide cannulae were inserted and protective caps were locked in place. Animals were allowed to recover for 1 week. Prior to all infusions, animals were lightly anesthetized (~2% isoflurane). The concentration of NMDA subunit antagonist AP5 (2-amino-5-phosphonopentanoate, AP-5) was 50 μM. Two microliters of AP-5 was infused bilaterally at a rate of 0.5 μL per minute. The dose remained unchanged for all animals across testing days. Following infusions, animals were allowed to fully recover in a clean cage (for 15 min on average) before behavioral testing began. After training finished brains were extracted, postfixed, and sectioned on a vibratome (Leica). Brightfield images were inspected under an upright microscope (Revolve Echo) and compared with a gerbil brain atlas (Radtke-Schuller et al., 2016) to verify the targeted striatal region.

### *In vitro* whole cell current clamp recordings

Recordings were digitized at 10 kHz on a Digidata 1550B (Molecular Devices) and analyzed offline (Axon pClamp 11). All recorded neurons had a resting potential ≤−50 mV and overshooting action potentials. Frequency-current (F-I) curves were constructed from the responses to 1,500 ms current pulses, in steps of 100 pA. Inhibitory postsynaptic potentials (IPSP) were evoked via biphasic stimulation of local fast-spiking interneurons (1–10 mV, 10 s interstimulus interval) in the presence of ionotropic glutamate receptor antagonists (6,7-Dinitroquinoxaline-2,3-dione, DNQX, 20 μM; 2-amino-5-phosphonopentanoate, AP-5, 50 μM) while held at −50 mV. The drugs were applied for a minimum of 8 min before recording IPSPs. Importantly, all recordings were systematically carried out at 200–300 microns from the right shank of the biphasic stimulator. To control for differences in stimulation strengths, we systematically employed 0.3–0.4 mA of stimulation to obtain a plateau in IPSP amplitudes. Once this maximum was reached, increasing stimulation did not lead to further increases in amplitude or duration of IPSPs. Excitatory post synaptic potentials (EPSPs) were evoked via biphasic stimulation of L5b ACx corticostriatal projection neurons in regular ACSF, while held at −80 mV to remove inhibitory potentials. To measure thresholds each cell was biphasically stimulated between 0.1 and 1.0 mA (0.1 mA, steps). The step at which an action potential was reliably measured was marked as the AP threshold. If the cell did not fire an action potential by 1.0 mA stimulation the threshold was marked as 1.0 mA to prevent damaging the L5b pyramidal cells. A modified theta burst stimulation (TBS) protocol was used to induce LTP (10 trains of 5 Hz; each train consisting of 10 pulses delivered over 2 s intervals; this was repeated 15 times at 10 s intervals). Prior to TBS, 10 sub-threshold baseline EPSPs were acquired at 30 s intervals using a stimulus intensity at which evoked EPSPs were ~50% of the AP threshold or 0.5 mA if threshold was not reached by 1.0 mA. During TBS, the same stimulus intensity was used and cells were held at −50 mV to facilitate spiking. Following TBS, cells were returned to their resting membrane potential for a few minutes and then held at −80 mV. The pre-TBS stimulus 50% threshold intensity was used to evoke 10 EPSPs at 30 s intervals every 5 min for 30 min. Data was acquired using a PC running Axon pClamp 11 data digitization software (Molecular Devices) and analyzed offline with Axon pClamp 11 macros.

### Statistical analyses

All statistical analyses and figure generation were carried out in JMP (SAS, Carey, NC, United States). Data was tested for normality and is displayed as mean ± SEM. For *in vivo* comparisons between more than two groups an ANOVA with Tukey HSD *post hoc* analysis was carried out. This test allows for the substantive variance in neural properties of single units between groups to be factored into the statistical significance testing across multiple behavioral epochs and between NH and HL groups. Comparison of *in vitro* data also used the ANOVA plus Tukey HSD *post hoc* analysis to compare across behavioral epochs and groups. For testing of behavioral performance (*d*-prime) over days, a MANOVA with linear regression analysis was used. Behavioral latency data used the ANOVA plus Tukey HSD *post hoc* analysis to compare across behavioral epochs and groups.

## Results

### Learning and behavioral performance did not differ between NH and HL groups

**Figure 1:**
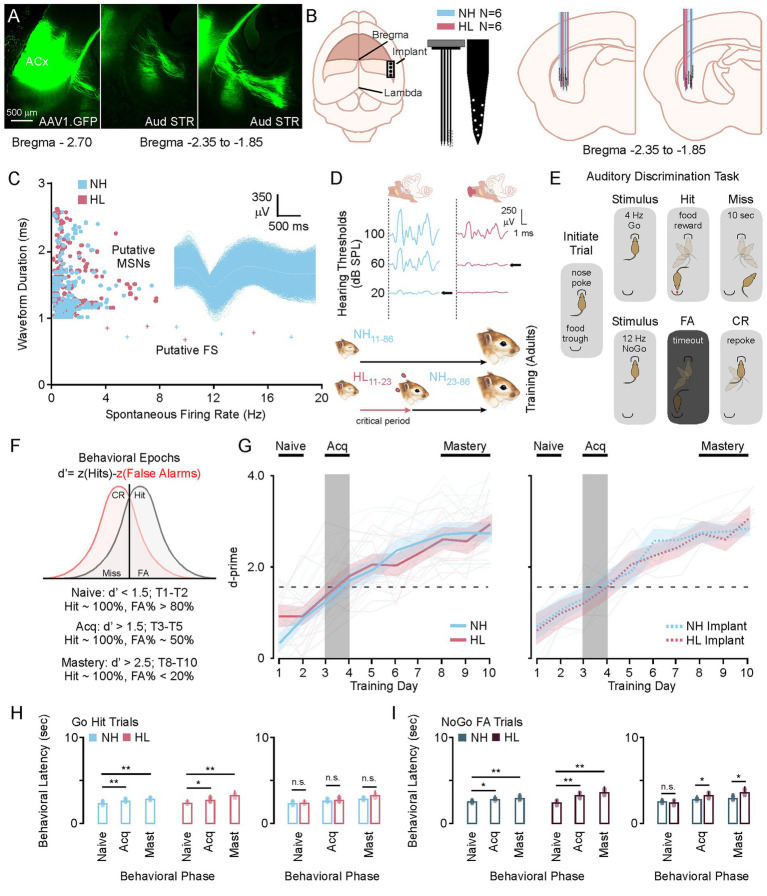
Phases of learning for the Amplitude Modulated Go No-Go Behavioral Task. **(A)** Photomicrographs showing an AAV tracer injection (AAV1.CamkII.GFP) into primary auditory cortex and subsequent labeling in the posterior tail of the striatum. **(B)** Cartoon showing the placement of the 64-channel electrode, and the relative positions of the recording sites recovered after histology. **(C)** Scatter plot showing the selection criterion for putative medium spiny neurons after PCA sorting, with an exemplar MSN waveform. **(D)** Auditory brainstem response data showing the sound attenuation produced by earplugging (top) and a diagram of the critical period hearing loss (bottom). **(E)** Diagram showing the behavioral paradigm. After nose poke a Go or NoGo auditory stimulus will play directing the animal to go to the trough or initiate a new trial with a re-poke. **(F)** Diagram showing how *d*-prime is calculated and how the behavioral epochs are determined by *d*-prime cutoffs. **(G)** Behavioral curves for the group average and individual animal data for normal hearing and hearing loss animals with and without electrode implants. **(H,I)** Bar plots showing the average and individual animal behavioral latency for Go and NoGo trials in normal hearing and hearing loss groups. ^*^p < 0.05, ^**^p < 0.01, and ^***^p < 0.001. FA, false alarm; CR, correct rejection; NH, normal hearing; HL, hearing loss; Acq, acquisition.

In this study we identified a region of the posterior striatum that receives dense innervation from auditory cortex (Figure 1A). We used this feedback to develop an approach for electrode implantation to record from medium spiny neurons in awake behaving animals. 64-channels electrode were implanted in this region in a total of 6 NH (3M/3F) and 6 HL (3M/3F) animals (Figure 1B). Offline PCA analysis with K-means sorting was used to identify putative medium spiny neurons based on waveform duration and firing rates (Figure 1C). Hearing loss was induced during the critical period of auditory development by inserting earplugs that attenuate sounds by ~35 dB SPL from P11 to P23 (Figure 1D). After this, earplugs were removed and animals experienced normal hearing throughout juvenile development and into adulthood (P23 to P86+). Normal hearing animals were sham earplugged (handled and ears manipulated) from P11 to P23 but otherwise had no manipulations to their hearing experience. Once animals reached adulthood at P86 they were trained on a Go NoGo auditory discrimination task and awake-behaving recordings were carried out during 10 days of testing (Figure 1E). Figure 1F shows the metric *d*-prime (*d*′ = z (H) − z (FA)), which was used to establish the criterion for task acquisition (*d*′ > 1.5 to < 2.5) and mastery (*d*′ > 2.5). Animals were considered in the naïve learning state when *d*′ was below 1.5 (T1–T2). The acquisition phase was based on previous work showing a brief window of learning around day 3–4 of this paradigm (Gay et al., 2023; Paraouty and Mowery, 2021; Sarro et al., 2015) and behavioral curves from 31 (15 NH, 16 HL) non-implanted animals (Figure 1G, left). Both the normal hearing group and the hearing loss group learned the task at roughly the same rates, achieving task acquisition between training day 3–4, and mastery by training day 9–10. Individual animal data is plotted to show the variance between animals across groups (transparent traces). There was no significant difference in the learning rate between the non-implanted NH and HL groups (*F*[1,29] = 1.43, *p* = 0.24). The implanted animals also showed no difference in learning rates between their non-implanted cohort for both normal hearing (*F*[1,19] = 016, *p* = 0.68) and hearing loss (*F*[1,20] = 0.56, *p* = 0.81) groups (Figure 1G, right). Furthermore, Figures 1H,I shows that there were no differences in latency to response for scoring a hit on the Go trials or a FA on the NoGo trials (Tukey HSD: behavioral latency mean ± SEM; Tables 1, 2). There were no significant differences between NH and HL animals across behavioral epochs for Go Trials; however, there were slightly significant increases in latency for the HL group during acquisition and mastery (Tukey HSD, behavioral latency mean ± SEM: Table 3).

**Table 1:** *In vivo* comparisons across behavioral epochs in NH and HL animals for Go Trials.

Group	Measure	Trial phase	Naive	Acq	Mastery	Naive vs. Acq	Naive vs. mastery	Acq vs. mastery
Normal hearing	Latency to hit (sec)	Trough arrival	2.53 ± 0.03	2.67 ± 0.02	2.92 ± 0.03	**, *p* < 0.01	**, *p* < 0.01	**, *p* < 0.01
	Latency to peak (ms)	Unmodulated sound	139 ± 27	248 ± 31	126 ± 31	***, *p* < 0.001	n.s. *p* > 0.1	***, *p* < 0.001
	Peak firing rate (%)	Unmodulated sound	25 ± 2	12 ± 2	23 ± 3	***, *p* < 0.001	n.s. *p* > 0.1	***, *p* < 0.001
	Latency to peak (ms)	Modulated sound	125 ± 10	143 ± 9	146 ± 9	**, *p* < 0.01	**, *p* < 0.01	n.s.
	Peak firing rate (%)	Trough arrival	54 ± 1.3	44 ± 1.2	60 ± 1.3	***, *p* < 0.001	n.s. *p* > 0.1	***, *p* < 0.001
Hearing loss	Latency to hit (sec)	Trough arrival	2.60 ± 0.04	2.81 ± 0.04	2.97 ± 0.03	*, *p* < 0.05	***, *p* < 0.001	*, *p* < 0.05
	Latency to peak (ms)	Unmodulated sound	119 ± 19	237 ± 27	107 ± 21	***, *p* < 0.001	n.s. *p* > 0.1	***, *p* < 0.001
	Peak firing rate (%)	Unmodulated sound	31 ± 4	15 ± 3	26 ± 3	***, *p* < 0.001	n.s. *p* > 0.1	***, *p* < 0.001
	Latency to peak (ms)	Modulated sound	117 ± 11	128 ± 10	131 ± 8	**, *p* < 0.01	**, *p* < 0.01	n.s.
	Peak firing rate (%)	Trough arrival	51 ± 1.4	37 ± 0.9	54 ± 1.2	***, *p* < 0.001	n.s. *p* > 0.1	***, *p* < 0.001

Acq, acquisition; sec, seconds; ms, milliseconds; n.s., not significant.

**Table 2:** *In vivo* comparisons across behavioral epochs in NH and HL animals for NoGo Trials.

Group	Measure	Trial phase	Naive	Acq	Mastery	Naive vs. Acq	Naive vs. mastery	Acq vs. mastery
Normal hearing	Latency to FA (sec)	Trough arrival	2.42 ± 0.07	2.79 ± 0.09	3.32 ± 0.17	*, *p* < 0.05	**, *p* < 0.01	**, *p* < 0.01
	Latency to peak (ms)	Unmodulated sound	157 ± 29	256 ± 3	154 ± 31	***, *p* < 0.001	n.s. *p* > 0.1	***, *p* < 0.001
	Peak firing rate (%)	Unmodulated sound	24 ± 3	13 ± 2	21 ± 3	***, *p* < 0.001	n.s. *p* > 0.1	***, *p* < 0.001
	Latency to peak (ms)	Modulated sound	33 ± 3	43 ± 3	49 ± 4	**, *p* < 0.01	**, *p* < 0.01	n.s. *p* > 0.1
	Peak firing rate (%)	Trough arrival	44 ± 1.0	33 ± 1.3	47 ± 1.1	***, *p* < 0.001	n.s. *p* > 0.1	***, *p* < 0.001
Hearing loss	Latency to FA (sec)	Trough arrival	2.48 ± 0.11	3.27 ± 0.13	3.65 ± 0.18	**, *p* < 0.01	**, *p* < 0.01	**, *p* < 0.01
	Latency to peak (ms)	Unmodulated sound	111 ± 27	233 ± 35	115 ± 32	***, *p* < 0.001	n.s. *p* > 0.1	***, *p* < 0.001
	Peak firing rate (%)	Unmodulated sound	26 ± 7	11 ± 5	23 ± 5	***, *p* < 0.001	n.s. *p* > 0.1	***, *p* < 0.001
	Latency to peak (ms)	Modulated sound	31 ± 3	39 ± 3	47 ± 4	**, *p* < 0.01	**, *p* < 0.01	n.s. *p* > 0.1
	Peak firing rate (%)	Trough arrival	41 ± 0.8	29 ± 0.7	42 ± 0.8	***, *p* < 0.001	n.s. *p* > 0.1	***, *p* < 0.001

Acq, acquisition; sec, seconds; ms, milliseconds; n.s., not significant; FA, false alarm.

**Table 3:** *In vivo* comparisons between NH and HL animals across behavioral epochs.

Trial typ	Measure	Trial phase	Naive NH vs. HL	Acq NH vs. HL	Mastery NH vs. HL	Naive vs. naive	Acq vs. Acq	Mast vs. mast
Go Trials	Latency to hit (sec)	Trough arrival	2.63 ± 0.03 vs. 2.60 ± 0.04	2.67 ± 0.02 vs. 2.81 ± 0.04	2.92 ± 0.03 vs. 2.97 ± 0.03	n.s. *p* > 0.1	n.s. *p* > 0.1	n.s. *p* > 0.1
	Latency to peak (ms)	Unmodulated sound	139 ± 27 vs. 119 ± 19	248 ± 31 vs. 237 ± 27	126 ± 31 vs. 107 ± 21	n.s. *p* > 0.1	n.s. *p* > 0.1	n.s. *p* > 0.1
	Peak firing rate (%)	Unmodulated sound	25 ± 2 vs. 31 ± 4	12 ± 2 vs. 15 ± 3	23 ± 3 vs. 26 ± 3	n.s. *p* > 0.1	n.s. *p* > 0.1	n.s. *p* > 0.1
	Latency to peak (ms)	Modulated sound	125 ± 10 vs. 117 ± 11	143 ± 9 vs. 128 ± 10	146 ± 9 vs. 131 ± 8	n.s. *p* > 0.1	n.s. *p* > 0.1	n.s. *p* > 0.1
	Peak firing rate (%)	Trough arrival	54 ± 13 vs. 51 ± 4	44 ± 12 vs. 37 ± 9	60 ± 13 vs. 54 ± 2	n.s. *p* > 0.1	n.s. *p* > 0.1	n.s. *p* > 0.1
NoGo Trials	Latency to FA (sec)	Trough arrival	2.42 ± 0.07 vs. 2.48 ± 0.11	2.79 ± 0.09 vs. 3.27 ± 0.13	3.32 ± 0.17 vs. 3.65 ± 0.18	n.s. *p* > 0.1	*, *p* < 0.05	*, *p* < 0.05
	Latency to peak (ms)	Unmodulated sound	167 ± 29 vs. 111 ± 27	256 ± 3 vs. 233 ± 35	164 ± 31 vs. 115 ± 32	**, *p* < 0.01	**, *p* < 0.01	**, *p* < 0.01
	Peak firing rate (%)	Unmodulated sound	24 ± 3 vs. 26 ± 7	13 ± 2 vs. 11 ± 5	21 ± 3 vs. 23 ± 5	n.s. *p* > 0.1	n.s. *p* > 0.1	n.s. *p* > 0.1
	Latency to peak (ms)	Modulated sound	134 ± 3 vs. 143 ± 3	143 ± 3 vs. 143 ± 3	143 ± 3 vs. 143 ± 3	n.s. *p* > 0.1	n.s. *p* > 0.1	n.s. *p* > 0.1
	Peak firing rate (%)	Trough arrival	44 ± 10 vs. 41 ± 8	34 ± 13 vs. 29 ± 7	47 ± 11 vs. 54 ± 2	n.s. *p* > 0.1	n.s. *p* > 0.1	n.s. *p* > 0.1

Acq, acquisition; sec, seconds; ms, milliseconds; n.s., not significant; FA, false alarm.

### Striatal population-level phase locking to auditory stimuli correlates with behavioral task acquisition

**Figure 2:**
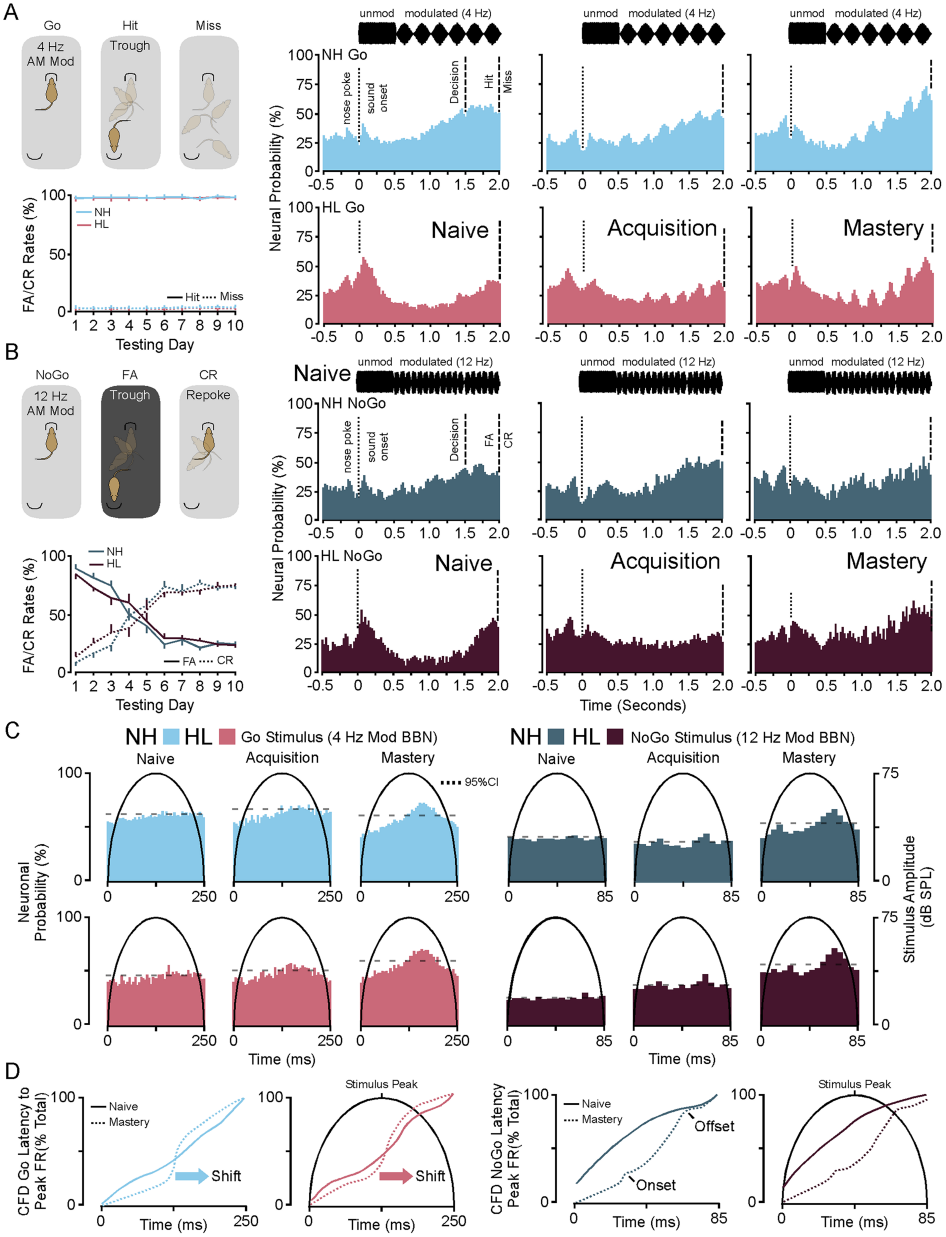
Neuronal stimulus response profiles during Go and NoGo Trials on the amplitude modulated discrimination task. **(A)** Diagram showing the behavioral response possibilities during a Go trial (top, left) and the average hit and miss percentages for implanted NH and HL animals over 10 days of training (bottom, left). (Right) Stimulus response profiles for Go trials during the three phases of learning for the normal hearing and hearing loss groups. **(B)** Cartoon showing the possible outcomes of the NoGo Trial (top, left) and the group average incidence rates of FA and CR for NH and HL animals over 10 days of training (bottom, left). (Right) Stimulus response profiles for NoGo trials during the three phases of learning for the normal hearing and hearing loss groups. **(C)** Plots showing the shifts in peak latency to firing for the 4 Hz Go stimulus (left) and the 12 Hz NoGo stimulus (right) throughout learning for the NH and HL groups. **(D)** Cumulative frequency distributions of the neural population data for latency to peak firing in Naïve versus mastery phases of learning for NH and HL groups during Go (left) and NoGo (right) trials. FA, false alarm; CR, correct rejection; NH, normal hearing; HL, hearing loss; Acq, acquisition.

For this study, recordings from 4,303 sound responsive putative single unit medium spiny neurons were analyzed during the naïve (T1–T2; *d*-prime <1.5), acquisition (~T3–T5; *d*-prime 1.5 to 2.0), and mastery (T9–T10; *d*-prime >2.5) phases of testing. Figure 2A shows a diagram of the Go Trial outcomes (left, top) and the hit/miss rates for all implanted animals (left, bottom). When an animal entered the food trough on a Go trial they received a food reward. Failure to visit the food trough after 10 s led to a miss and the availability of the next trial. Figure 2A (right) shows the group averaged stimulus response profiles (25 ms bins) for the neural population responses to nose poke, sound onset (unmodulated), and Go stimulus (4 Hz modulated) during Go Trials for the NH and HL groups. The stimulus response clearly emerges during acquisition and is refined and increased by mastery of the task. Figure 2B shows a diagram of the NoGo Trial outcomes (left, top) and the false alarm/correct rejection rates for all implanted animals (left, bottom). On these trials the animals had two options. They could either go to the food trough to score a false alarm and a time out (5 s of darkness and lack of trial initiation) or they could repoke and immediately initiate a Go Trial. Figure 2B (right) shows the group averaged stimulus response profiles (25 ms bins) for the neural population responses to nose poke, sound onset (unmodulated), and NoGo stimulus (12 Hz modulated) during NoGo Trials for the NH and HL groups. Here you can see that phase locking begins in the acquisition epoch and becomes robust during mastery.

Figure 2C (left) shows the averaged peak response for each stimulus modulation in the normal hearing and hearing loss group at each behavioral epoch during the Go Hit trials. There is a clear alignment and shift of the peak that moves slightly to the right of the peak amplitude of the modulation for both groups as the task is mastered. Figure 2C (right) shows the averaged peak response for each stimulus modulation in the normal hearing and hearing lost group at each behavioral epoch during the NoGo false alarm trials. Unlike the Go stimulus two peaks of activation occur left and right of the peak amplitude of the modulation with the more prominent latency to peak occurring to the right of the stimulus amplitude peak. Note that three NoGo stimulus sweeps occur (~83.3 ms) for every single Go stimulus (250 ms).

In Figure 2D (left) a cumulative frequency distribution for each medium spiny neuron illustrates the shift from randomized peaks (naïve) to alignment to the right of the peak of the modulation for both groups. For all medium spiny neurons there are significant increases in the average peak latency between naïve and acquisition and naïve and mastery phases of learning for both normal hearing and hearing loss groups (Tukey HSD, latency to peak mean ± SEM, *p*-value; Table 1). There were no significant differences between NH and HL animals across behavioral epochs (Tukey HSD, latency to peak mean ± SEM, *p*-value; Table 3). In Figure 2D (right) a cumulative frequency distribution for each medium spiny neuron illustrates the shift from randomized peaks (naïve) to a small number of cells aligning with the onset of the stimulus and most cells aligning with the waning of the stimulus for both groups. Again, for all medium spiny neurons there are significant increases in the average peak latency between naïve and acquisition and naïve and mastery phases of learning for both normal hearing and hearing loss groups (Tukey HSD, latency to peak mean ± SEM, *p*-value; Table 2). There were no significant differences between NH and HL animals across behavioral epochs (Tukey HSD: latency to peak mean ± SEM, *p*-value; Table 3).

**Figure 3:**
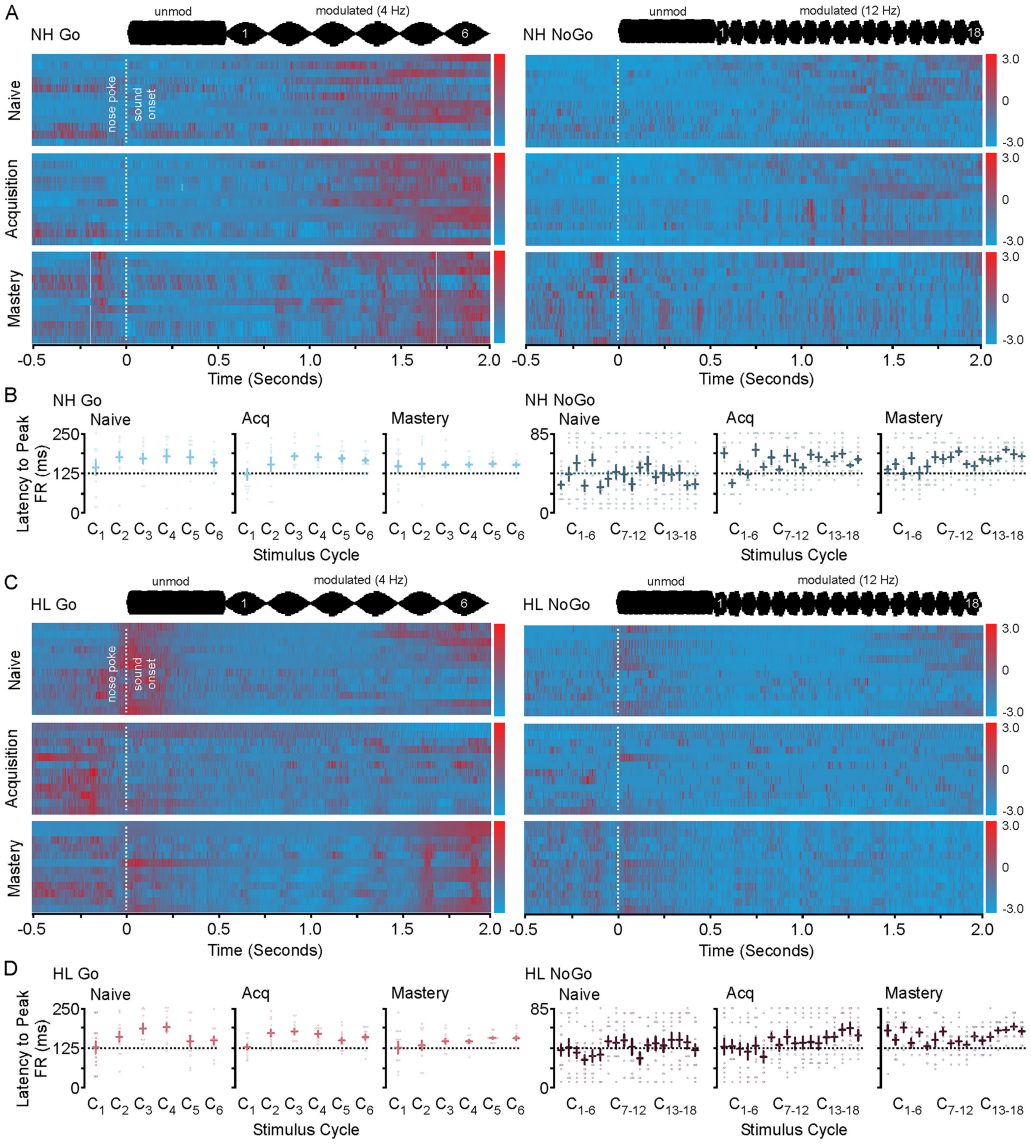
Heat maps for NH and HL animals during Go and NoGo trials for each behavioral epoch. **(A)** Heat map showing the changes to sound induced neural activity during go (left) and No-Go (right) trials in normal hearing animals as they progress from naive to mastery of the task. In each heat map the 2 days of data used in analysis for each animal for each epoch are displayed. **(B)** Scatter plots showing the latency to peak firing rates for all animals in the NH group during Go (left) and NoGo (right) trials across behavioral epochs. **(C)** Heat map showing the changes to sound induced neural activity during go (left) and No-Go (right) trials in hearing loss animals as they progress from naive to mastery of the task. In each heat map the 2 days of data used in analysis for each animal for each epoch are displayed. **(D)** Scatter plots showing the latency to peak firing rates for all animals in the HL group during Go (left) and NoGo (right) trials across behavioral epochs.

Figure 3 shows heat maps for each animals’ neural activity throughout training. Each heat map has been constructed by standardizing the average neural activity for each animal on the 2 days of recordings used per each behavioral epoch. Thus, represented is the T1/T2 (naïve), T3/T4 (acquisition), and T9/T10 (mastery) data. In Figure 3A the heat maps for the NH group during Go and NoGo trials across behavioral epochs are shown. Figure 3B shows the average latency to peak firing rate for all NH animals during each stimulus cycle and behavioral phase for the Go (left) and NoGo (right) trials. Here the means are the same across behavioral epochs; however, the variance decreases towards the phase locked average just right of the peak of the stimulus for the Go Trials (Levene *F* = 8.4, *p* < 0.001) and the NoGo Trials (Levene *F* = 3.3, *p* < 0.001) on later cycles 4–6. In Figure 3C the heat maps for the HL group during Go and NoGo trials across learning are shown. Figure 3D shows the average latency to peak firing rate for all HL animals during each stimulus cycle and behavioral phase for the Go (left) and NoGo (right) trials. Again, the means are the same across behavioral epochs; however, the variance decreases towards the phase locked average just right of the peak of the stimulus for the Go Trials (Levene *F* = 2.0, *p* = 0.024, *p* < 0.05) and the NoGo Trials (Levene *F* = 2.6, *p* = 0.008, *p* < 0.05); especially for the later cycles of the stimulus. In both the Go and the NoGo trials for each group you can see the emergence of the phase locking to the stimulus rate (Go 4 Hz, No-Go 12 Hz). When viewed in this way, the neural responses and phase locking to the AM stimuli initially occur near the time of reward or lack of reward during task acquisition, but then shifts towards the onset of the modulated signal as the animal masters the task.

### Significant changes to neural activity and latency to peak firing rate accompany behavioral acquisition

**Figure 4:**
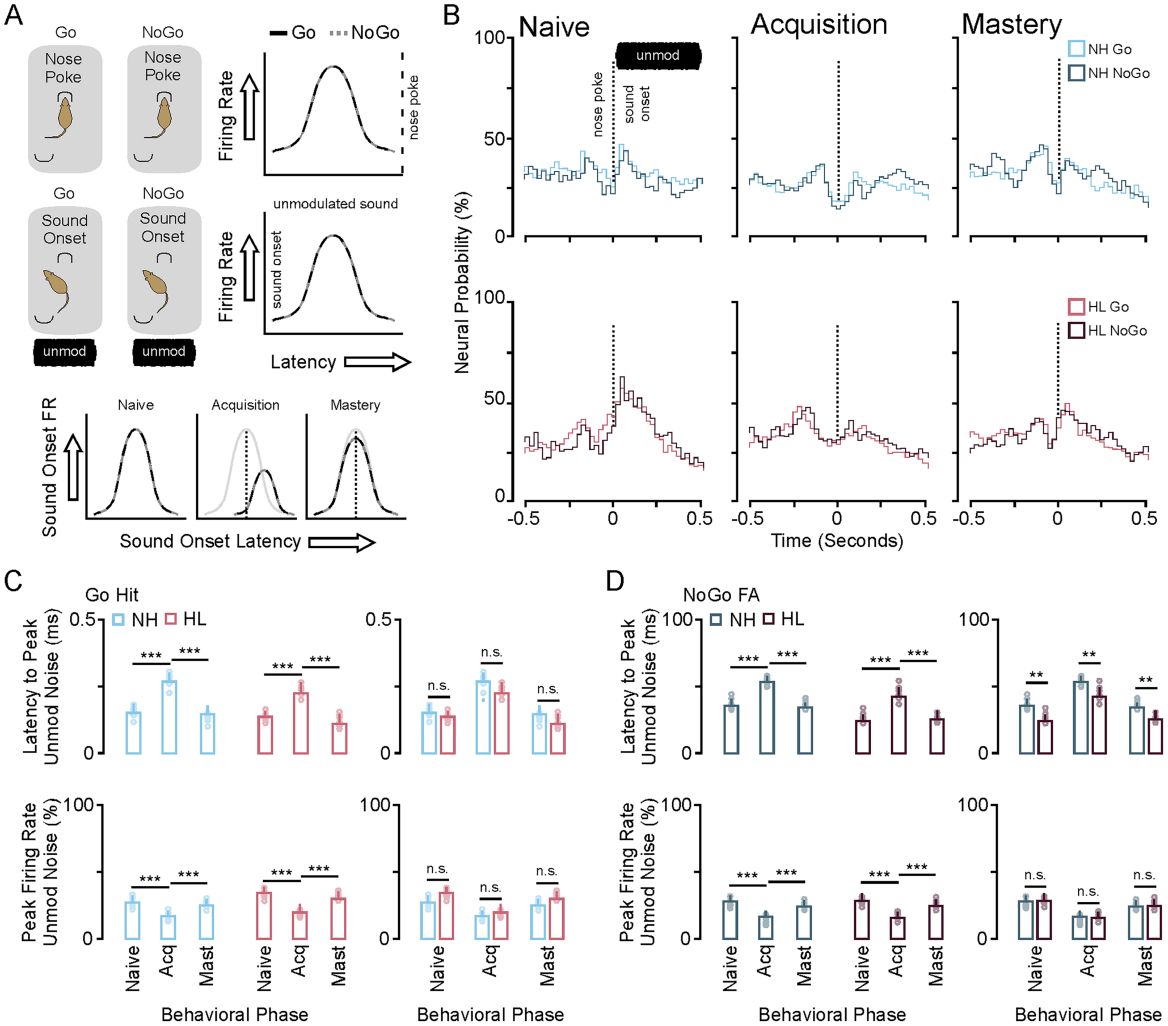
Changes in neural population response to nose poke and unmodulated sound during task acquisition. **(A)** Cartoon showing that the Go and NoGo trial are approximately the same prior to modulated sound onset. As such the neural response to the nosepoke and non-modulated sound onset is the same across Go and NoGo trials (right). Neural suppression and increased latency to peak firing occur during task acquisition for both trial types (bottom). **(B)** Stimulus response profiles for Go (color) and NoGo (grey) trials during the three phases of learning for the NH and HL groups. **(C)** Bar plots showing the group average and individual animal data for changes in latency to peak (top) and peak firing rates (bottom) across learning for the Go and NoGo trials in the NH group. **(D)** Bar plots showing the group average and individual animal data for changes in latency to peak (top) and peak firing rates (bottom) across learning for the Go and NoGo trials in the HL group. ^*^p < 0.05, ^**^p < 0.01, and ^***^p < 0.001. FA, false alarm; CR, correct rejection; NH, normal hearing; HL, hearing loss; Acq, acquisition.

Figure 4 shows neural firing rate and latency to peak firing rates during the nose poke and onset of unmodulated sound. An interesting feature of this task is that the animal does not know what type of trial has been initiated until the modulated sound stimulus begins 500 ms after nose poke (Figure 3A). During the acquisition phase there is no difference between the neural activity surrounding the nose poke or the onset of the non-modulated sound stimulus. There is a significant shift in latency and decrease in neural activity during acquisition for the neural response to the onset to sound. This can be seen in the group averaged stimulus response profiles (25 ms bins) for the neural population responses to nose poke, sound onset (unmodulated) for the Go and NoGo trials for both NH and HL groups (Figure 4B). Figure 4C shows the significant increase in latency for unmodulated sound onset and decrease in firing rate for the NH and HL groups during Go Trials for each behavioral epoch (Tukey HSD: mean ± SEM; Table 1). Figure 4D shows the significant increase in latency for unmodulated sound onset and decrease in neural activity for the NH and HL groups during NoGo Trials for each behavioral epoch (Tukey HSD: latency to peak mean ± SEM, *p*-value; Table 2). There were significant differences between NH and HL animals across behavioral epochs (Tukey HSD: latency to peak mean ± SEM, *p*-value; Table 3).

### A transient reduction to neuronal population response to reward (hit) and no reward (false alarm) coincide with behavioral acquisition

**Figure 5:**
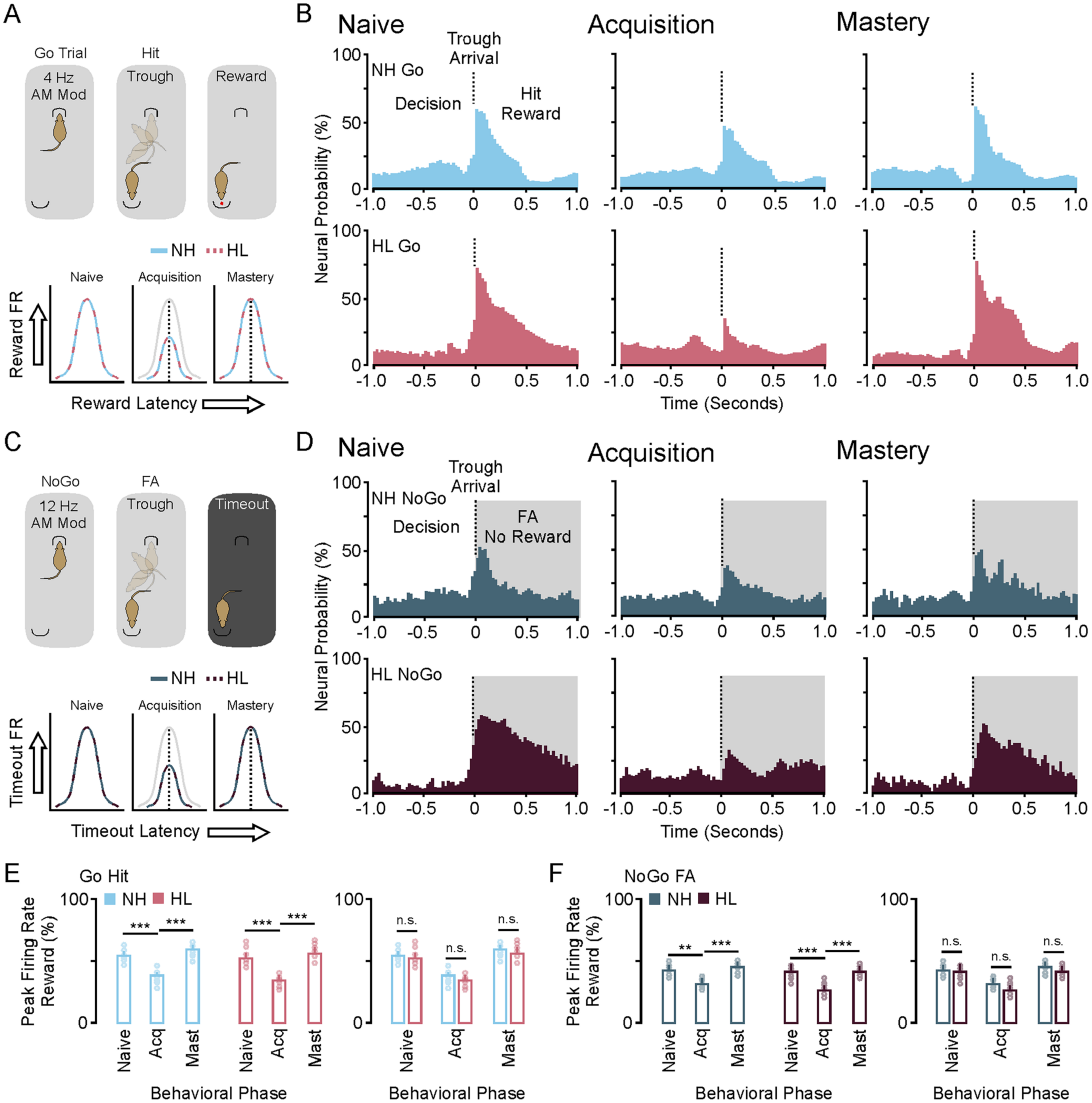
Changes in neural population response to reward and punishment during task acquisition. **(A)** Cartoon showing the changes in neural activity to the reward in the Go trial are suppressed during task acquisition for both groups. **(B)** Stimulus response profiles for Go trials during the three phases of learning for the NH and HL groups. **(C)** Cartoon showing the changes in neural activity to the punishment (timeout) in the No-Go FA trial are suppressed during task acquisition for both groups. **(D)** Stimulus response profiles for NoGo trials during the three phases of learning for the NH and HL groups. **(E)** Scattergrams showing the mean average and individual population data for peak firing rate to the reward during the Go Hit trial for both the NH and HL groups. **(F)** Scattergrams showing the mean average and individual population data for peak firing rate to the punishment during the No-Go FA trial for both the NH and HL groups. ^*^p < 0.05, ^**^p < 0.01, and ^***^p < 0.001. FA, false alarm; CR, correct rejection; NH, normal hearing; HL, hearing loss; Acq, acquisition.

Figure 5 shows the neural response to both the food reward during a Go Hit Trial and the lack of a food reward and timeout associated with the No-Go false alarm trial. On each Go trial the animal has the option to go to the food trough where they will receive a food pellet (Figure 5A). During the acquisition phase the neural response is significantly reduced for both the NH and HL groups. This can be seen in the group averaged stimulus response profiles (25 ms bins) for the neural population responses to reward for both groups (Figure 5B). During the acquisition phase there is a clear reduction in the firing rate after receiving the food reward in both groups. On each NoGo trial the animal has the option to go to the food trough where they will receive only a timeout punishment (Figure 5C). Like the Go Trial, during the acquisition phase the neural response is significantly reduced for both the NH and HL groups. For the group averaged stimulus response profiles (25 ms bins) the neural population responses to timeout for both groups show a clear reduction in firing rate (Figure 5D). Comparison between the Go and NoGo Trial population responses shows increases in neural activity (outside of learning) regardless of whether a food reward is given. Figure 5E shows changes to each medium spiny neurons peak firing rate during the Go Trial for both the NH and HL group. There is a significant overall reduction in firing rates for all sound responsive neurons during the Go trials for both groups (Tukey HSD: firing rate mean ± SEM, *p*-value; Tables 1, 2). Figure 5F shows changes to each medium spiny neurons peak firing rate during the NoGo Trial for both the NH and HL group. Again, there is a significant overall reduction in firing rates for all sound responsive neurons during the NoGo trials for both groups (Tukey HSD: firing rate mean ± SEM, *p*-value; Tables 1, 2). There were no significant differences between NH and HL animals across behavioral epochs (Tukey HSD: firing rate mean ± SEM, *p*-value; Table 3).

### Transient shifts in E/I tone create a brief window of plasticity that supports long term potentiation

**Figure 6:**
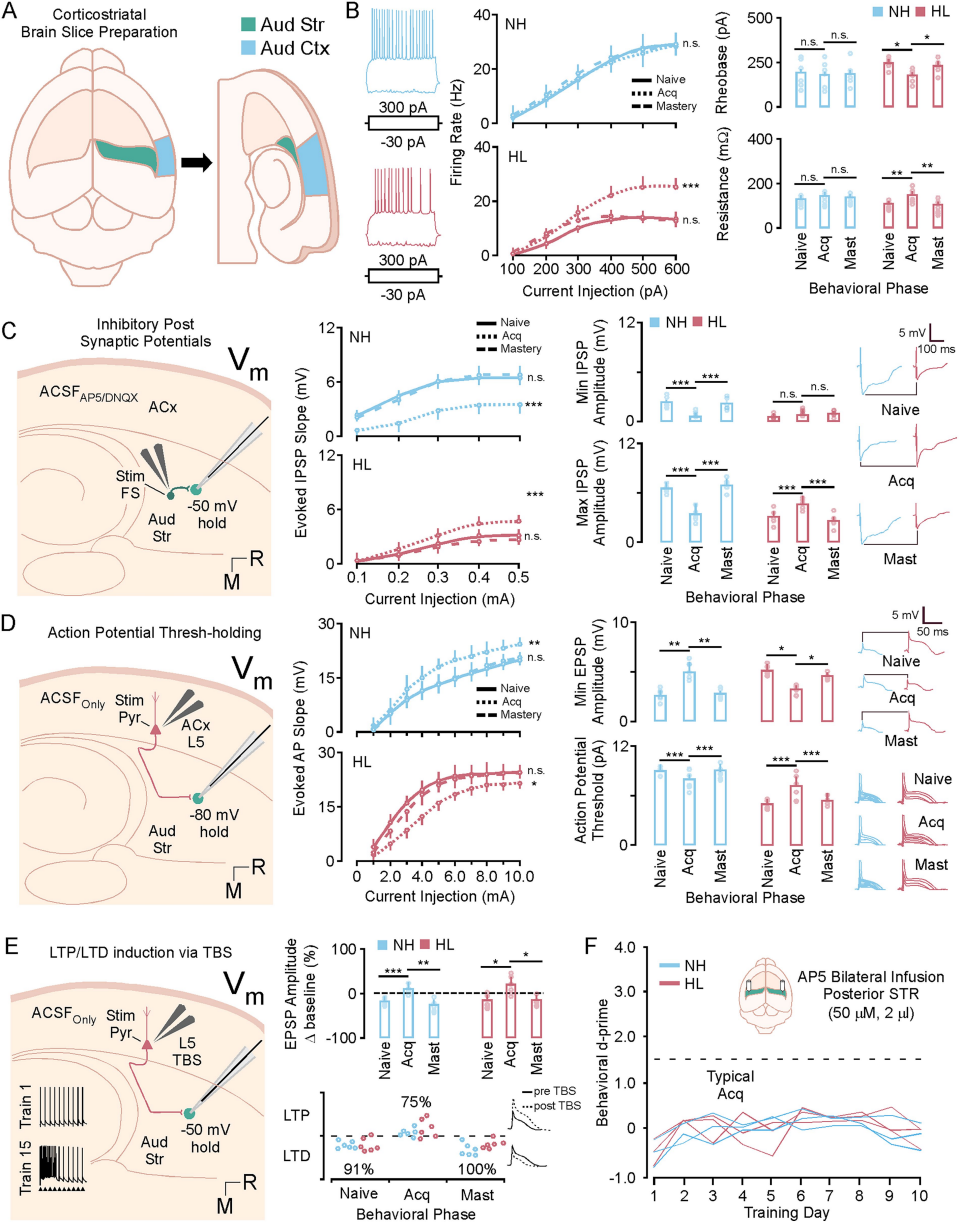
A brief window of synaptic and intrinsic plasticity during task acquisition. **(A)** Diagram showing the corticostriatal slice preparation. **(B)** Left shows representative examples of a medium spiny neuron evoked response to 300 pa and −30 pA. Middle, shows line plots of the F/I curves for the NH and HL group over behavioral phases. Right, top shows bar plots of the NH and HL group averages for rheobase over behavioral phases. Right, bottom shows bar plots of the NH and HL group averages for resistance over behavioral phases. **(C)** Shows a diagram of the slice configuration for recording IPSPs *in vitro* (left) Middle shows line plots of the IPSP slopes for the NH and HL group over behavioral phases. Right, top shows bar plots of the NH and HL group averages for min evoked IPSP amplitudes over behavioral phases. Right, bottom shows bar plots of the NH and HL group averages for max evoked IPSP amplitudes over behavioral phases. **(D)** Shows a diagram of the slice configuration for recording EPSPs *in vitro* (left). Middle shows line plots of the EPSP slopes for the NH and HL group over behavioral phases. Right, top shows bar plots of the NH and HL group averages for min evoked IPSP amplitudes over behavioral phases. Right, bottom shows bar plots of the NH and HL group averages for action potential (AP) thresholds over behavioral phases. **(E)** Shows a diagram for the configuration for theta burst induced LTP in the slice preparation (left). Middle shows bar plots for the mean average potentiation data for NH and HL animals over behavioral phases (top) and the individual animal data for LTP and LTD expression (bottom). **(F)** Behavioral data showing *d*-prime data for NH and HL animals over 10 days of training that received daily cannula infusions of NMDA blocker (AP-5, 50 mM, 2 mL). ^*^p < 0.05, ^**^p < 0.01, and ^***^p < 0.001. LTP, long term potentiation; LTD, long term depression; Vm, resting voltage; TBS, theta burst stimulation; IPSP, inhibitory post synaptic potential; EPSP, excitatory post synaptic potential; NH, normal hearing; HL, hearing loss; pA, pico amps; mV, millivolts; M, medial; R, rostral; aud str, auditory striatum; aud ctx, auditory cortex; ACSF, artificial cerebrospinal fluid.

Figure 6 shows *in vitro* whole cell recording data taken from animals that were learning the task. In these experiments 72 adult animals were trained on the task. Both normal hearing (18M/18F) and hearing loss (18M/18F) animals were used. After each day of training the *d*-prime was calculated and animals that met the criteria for the naïve (*d*′ < 1.5), acquisition (*d*′ > 1.5 to < 2.5), and mastery behavioral epoch (*d*′ > 2.5) were randomly selected to undergo corticostriatal slice preparation (Figure 6A). After verifying that the cell had a healthy resting potential (at least −50 mV) intrinsic data was collected for each cell. Figure 6B shows intrinsic firing properties, rheobase, and resistance for each cell divided by behavioral epoch for each group. Representative examples of a medium spiny neuron evoked response to 300 pa and −30 pA is shown for the NH and HL group (left). Figure 6B, middle shows input output functions for medium spiny neurons in the NH and HL group during learning. For NH animals comparison across the behavioral epoch shows no significant change in firing curves at any point in learning; however, firing rates return to normal physiological levels in the HL group during behavioral acquisition (MANOVA regression: F/I slope; Table 4). There was a significantly lower baseline firing rate induced by the developmental hearing loss that led to significant differences in firing rates between NH and HL animals in naïve and mastery epoch animals (MANOVA regression: F/I slope; Table 5). Figure 6B right, top shows average rheobase data for normal hearing and hearing loss groups across behavioral epochs. The NH group shows no changes; however, the hearing loss groups higher rheobase is significantly reduced during acquisition while firing rates increase to near normal levels (Tukey HSD; rheobase mean ± SEM; *p*-value; Table 4). Furthermore, there were significant differences in rheobase between the NH and HL group at baseline and after learning but not during acquisition (Tukey HSD; resistance mean ± SEM; *p*-value; Table 5). Figure 6B right, bottom shows average membrane resistance data for normal hearing and hearing loss groups across behavioral epochs. The NH group shows no changes; however, the hearing loss groups lower resistance is significantly increased during acquisition, again at the same time that firing rates increase to near normal levels (Tukey HSD; resistance mean ± SEM; *p*-value; Table 4). There were significant differences between the NH and HL group at baseline and after learning but not during acquisition (Tukey HSD; resistance mean ± SEM; *p*-value; Table 5). For resting membrane potential (RMP) the NH group shows no changes; however, the hearing loss groups hyperpolarized membrane becomes briefly more depolarized to NH levels during task acquisition. This lowers the inflection point faciliting action potential generation (Tukey HSD; RMP mean ± SEM; *p*-value; Table 4). Again there were significant differences between the NH and HL group at baseline and after learning but not during the acquisition phase (Tukey HSD; RMP mean ± SEM; *p*-value; Table 5).

**Table 4:** *In vitro* physiology comparisons across behavioral epochs in NH and HL animals.

Group	Measure	Naive	Acq	Mastery	Naive vs. Acq	Naive vs. mastery	Acq vs. mastery
Normal hearing	Firing rate (slope)	*F*[1,83] = 1.2	*F*[1,77] = 0.31	*F*[1,84] = 0.84	n.s. *p* > 0.1	n.s. *p* > 0.1	n.s. *p* > 0.1
	Rheobase (pA)	196 ± 12	181 ± 10	188 ± 16	n.s. *p* > 0.1	n.s. *p* > 0.1	n.s. *p* > 0.1
	Resistance (mOhms)	134 ± 6.9	147 ± 6.7	143 ± 8.1	n.s. *p* > 0.1	n.s. *p* > 0.1	n.s. *p* > 0.1
	RMP (mV)	−63 ± 0.59	−63 ± 0,77	−64 ± 0.63	n.s. *p* > 0.1	n.s. *p* > 0.1	n.s. *p* > 0.1
	IPSP slope (mV)	*F*[1,49] = 101	*F*[1,44] = 0.073	*F*[1,49] = 73.1	***, *p* < 0.001	n.s. *p* > 0.1	***, *p* < 0.001
	IPSP min amplitude (mV)	2.47 ± 0.82	0.78 ± 0.62	2.29 ± 1.01	***, *p* < 0.001	n.s. *p* > 0.1	***, *p* < 0.001
	IPSP max amplitude (mV)	6.63 ± 0.25	3.58 ± 0.20	6.97 ± 0.34	***, *p* < 0.001	n.s. *p* > 0.1	***, *p* < 0.001
	EPSP slope (mV)	*F*[1,34] = 9.14	*F*[1,33] = 0.55	*F*[1,35] = 8.33	**, *p* < 0.01	n.s. *p* > 0.1	**, *p* < 0.01
	EPSP min amplitude (mV)	2.70 ± 0.45	5.04 ± 0.42	2.81 ± 0.44	**, *p* < 0.01	n.s. *p* > 0.1	**, *p* < 0.01
	AP threshold (pA)	9.97 ± 0.32	7.84 ± 0.57	9.94 ± 0.34	**, *p* < 0.01	n.s. *p* > 0.1	**, *p* < 0.01
	Potentiation (%)	−30.2 ± 0.3	18.4 ± 0.7	−24.0 ± 0.4	***, *p* < 0.001	n.s. *p* > 0.1	**, *p* < 0.01
Hearing loss	Firing rate (slope)	*F*[1,81] = 40.1	*F*[1,75] = 0.91	*F*[1,79] = 20.2	***, *p* < 0.001	n.s. *p* > 0.1	***, *p* < 0.001
	Rheobase (pA)	245 ± 15	181 ± 10	233 ± 18	*, *p* < 0.05	n.s. *p* > 0.1	*, *p* < 0.05
	Resistance (mOhms)	113 ± 5.7	153 ± 11.2	109 ± 7.8	**, *p* < 0.01	n.s. *p* > 0.1	**, *p* < 0.01
	RMP (mV)	−67 ± 0.46	−65 ± 0.53	−68 ± 0.49	**, *p* < 0.01	n.s. *p* > 0.1	*, *p* < 0.05
	IPSP slope	*F*[1,48] = 14.2	*F*[1,42] = 1.32	*F*[1,48] = 24.4	***, *p* < 0.001	n.s. *p* > 0.1	***, *p* < 0.001
	IPSP min amplitude (mV)	0.82 ± 0.52	0.81 ± 0.48	1.09 ± 0.56	n.s. *p* > 0.1	n.s. *p* > 0.1	n.s. *p* > 0.1
	IPSP max amplitude (mV)	3.21 ± 0.25	4.73 ± 0.22	2.68 ± 0.25	***, *p* < 0.001	n.s. *p* > 0.1	*, *p* < 0.001
	EPSP slope (mV)	*F*[1,33] = 7.03	*F*[1,33] = 0.55	*F*[1,31] = 0.78	*, *p* < 0.05	n.s. *p* > 0.1	**, *p* < 0.05
	EPSP min amplitude (mV)	5.22 ± 0.46	3.33 ± 0.42	4.60 ± 0.45	**, *p* < 0.01	n.s. *p* > 0.1	*, *p* < 0.05
	AP threshold (pA)	4.68 ± 0.28	7.15 ± 0.42	5.23 ± 0.30	**, *p* < 0.01	n.s. *p* > 0.1	*, *p* < 0.05
	Potentiation (%)	−26.7 ± 0.5	7.1 ± 0.8	−27.3 ± 0.6	*, *p* < 0.05	n.s. *p* > 0.1	*, *p* < 0.05

Acq, acquisition; sec, seconds; ms, milliseconds; n.s., not significant; FA, false alarm; NH, normal hearing; HL, hearing loss; mast, mastery; IPSP, inhibitory post synaptic potential; AP, action potential; LTP, long term potentiation; mV, millivolts; pA, picoamps.

**Table 5:** *In vitro* comparisons between NH and HL animals across behavioral epochs.

Measure	Naive NH vs. HL	Acq NH vs. HL	Mastery NH vs. HL	Naive vs. naive	Acq vs. Acq	Mast vs. mast
Firing rate (slope)	0.82 ± 0.16 vs. 0.74 ± 0.05	0.96 ± 0.12 vs. 1.14 ± 0.16	1.12 ± 0.12 vs. 0.77 ± 0.11	***, *p* < 0.001	n.s. *p* > 0.1	***, *p* < 0.001
Rheobase (pA)	196 ± 12 vs. 248 ± 15	181 ± 13 vs. 181 ± 10	188 ± 16 vs. 233 ± 18	*, *p* < 0.05	n.s. *p* > 0.1	*, *p* < 0.05
Resistance (mOhms)	134 ± 6.9 vs. 113 ± 5.7	147 ± 6.7 vs. 153 ± 11.2	143 ± 8.1 vs. 109 ± 7.8	*, *p* < 0.05	n.s. *p* > 0.1	*, *p* < 0.05
RMP (mV)	−63 ± 0.59 vs. −67 ± 0.46	-63 ± 0.77 v − 65 ± 0.53	−64 ± 0.63 vs. −68 ± 0.49	**, *p* < 0.01	n.s. *p* > 0.05	**, *p* < 0.01
IPSP slope (mV)	*F*[1,43] = 125	*F*[1,54] = 2.7	*F*[1,43] = 101	***, *p* < 0.001	n.s. *p* > 0.1	***, *p* < 0.001
IPSP min amplitude (mV)	2.47 ± 0.82 vs. 0.82 ± 0.52	0.78 ± 0.62 vs. 0.81 ± 0.48	2.29 ± 1.01 vs. 1.09 ± 0.56	***, *p* < 0.001	n.s. *p* > 0.1	***, *p* < 0.001
IPSP max amplitude (mV)	6.63 ± 0.25 vs. 3.21 ± 0.25	3.58 ± 0.20 vs. 4.73 ± 0.22	6.97 ± 0.34 vs. 2.68 ± 0.25	***, *p* < 0.001	n.s. *p* > 0.1	***, *p* < 0.001
EPSP slope (mV)	*F*[1,31] = 27.5	*F*[1,36] = 0.051	*F*[1,33] = 19.5	***, *p* < 0.001	n.s. *p* > 0.1	***, *p* < 0.001
EPSP min amplitude (mV)	2.71 ± 0.45 vs. 5.20 ± 0.46	5.04 ± 0.42 vs. 3.33 ± 0.42	2.80 ± 0.44 vs. 4.60 ± 0.45	*, *p* < 0.05	n.s. *p* > 0.1	*, *p* < 0.05
AP threshold (pA)	9.97 ± 0.32 vs. 4.68 ± 0.28	7.84 ± 0.57 vs. 7.15 ± 0.42	9.94 ± 0.34 vs. 5.23 ± 0.30	***, *p* < 0.001	n.s. *p* > 0.1	***, *p* < 0.001
Potentiation (%)	−30.2 ± 0.3 vs. −26.7 ± 0.5	18.4 ± 0.7 vs. 7.1 ± 0.8	−24.0 ± 0.4 vs. −27.3 ± 0.6	n.s. *p* > 0.1	n.s. *p* > 0.1	n.s. *p* > 0.1

Acq, acquisition; sec, seconds; ms, milliseconds; n.s., not significant; FA, false alarm; NH, normal hearing; HL, hearing loss; mast, mastery; IPSP, inhibitory post synaptic potential; AP, action potential; LTP, long term potentiation; mV, millivolts; pA, picoamps.

For 36 male and female animals (18 NH, 18 HL) inhibitory post synaptic potentials were collected by adding AMPA and NMDA blocker to the solution and holding the cells at −50 m during local biphasic stimulation which activated local fast spiking interneurons (Figure 6C). Figure 6C middle shows the IPSP slopes for increasing biphasic stimulation of the local striatal FS cells. For the Normal hearing group there is a significant reduction in slope during acquisition and for the HL group there is a significant increase in slope during acquisition (MANOVA regression: F/I slope; Table 4). Figure 6C right shows min (top) and max (bottom) evoked IPSP data for the NH and HL groups and representative examples of max evoked IPSPs at each behavioral epoch. There is a significant decrease for NH animals evoked IPSP min and max and a significant increase for the HL animals evoked IPSP min and max during the acquisition phase (Tukey HSD; IPSP min, IPSP max, mean ± SEM; *p*-value; Table 4). There are also significant differences between NH and HL animals at baseline, but not during acquisition (Tukey HSD; mean ± SEM; *p*-value; Table 5).

For 36 male and female animals (18 NH, 18 HL) excitatory thresholds were collected by holding the cells at −80 mV and biphasically stimulating L5 excitatory inputs (Figure 6D). Figure 6D middle shows the EPSP slopes for increasing biphasic stimulation of the L5 ACx inputs. For the Normal hearing group there is a significant increase and for the HL group there is a significant decrease in slope during acquisition (MANOVA regression: EPSP slope mean ± SEM, *p*-value; Table 4). There were significant differences between NH and HL animals at baseline, but not during acquisition (MANOVA regression: EPSP slope; Table 4). Figure 6D right shows min evoked EPSP (top) and AP threshold (bottom) data for the NH and HL groups and representative examples of min evoked EPSPs and AP thresholds at each behavioral epoch. AP threshold was determined by increasing voltage (0.1 mA steps) to determine the inflection points of the action potential for each cell. There is a significant increase in NH animals’ min evoked EPSP and decrease in AP threshold and a significant decrease in HL animals’ min evoked EPSP and increase in AP threshold during behavioral acquisition (Tukey HSD; EPSP min, AP threshold mean ± SEM; *p*-value; Table 4). There are also significant differences between NH and HL animals at baseline, but not during acquisition (Tukey HSD; EPSP min, AP threshold, mean ± SEM; *p*-value; Table 5).

After establishing an excitatory threshold for these cells, they were run through a theta burst protocol (see methods; Figure 6E, left). This was carried out at 50% of the max current required to elicit an AP while the cells were held at −50 mV (closer to AP threshold). Following TBS protocol EPSPs were recorded by continuing to biphasically stimulate the medium spiny neurons at 50% max threshold. Ten recordings were taken every 5 min for 30 min and the % difference from pre-TBS amplitude for the 30-min recording was quantified (Figure 6E, middle, top). This shows that during acquisition medium spiny cells in both the NH and the HL group are significantly more likely to undergo LTP (Tukey HSD: TBS potentiation mean ± SEM; *p*-value; Table 4). In this case, the potentiation shifts were not different between the NH and HL groups (Tukey HSD: TBS potentiation mean ± SEM; *p*-value; Table 5). Figure 6E middle, bottom shows the individual changes to EPSP amplitude for each animal across behavioral epochs and representative examples of LTP vs. LTD. Figure 6F shows *in vivo* cannula infusion experiments, in which NMDA receptor activity in the posterior tale of the striatum was blocked with AP-5. Daily infusions of NMDA blocker prevented behavioral acquisition of the task for both NH and HL groups (MANOVA regression: *d*-prime *F*[1,4] = 1.53, *p* > 0.1).

## Discussion

**Figure 7:**
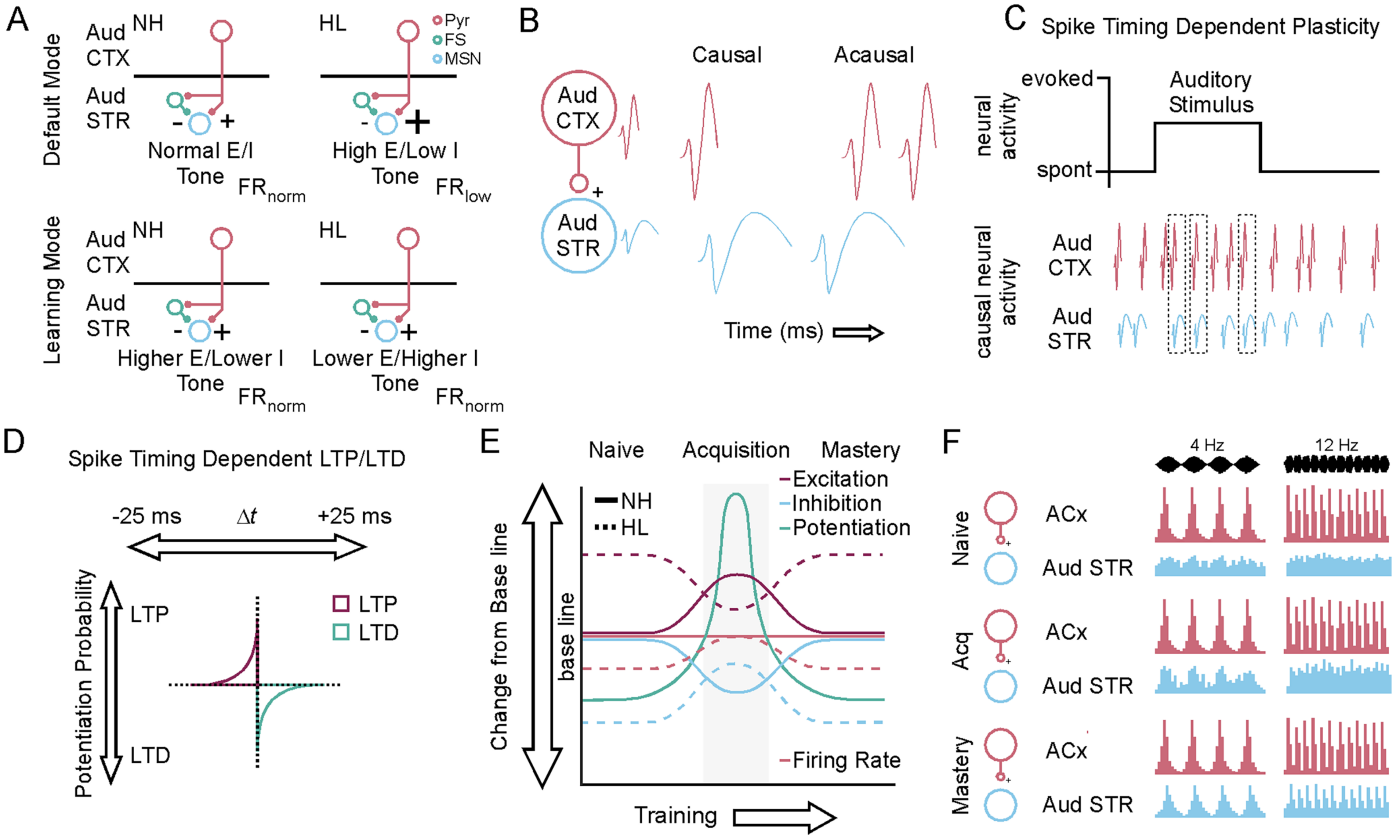
A brief window of plasticity supports the emergence of neural population responses to conditioned auditory stimuli. **(A)** Diagram showing the baseline differences between NH and HL animals E/I tone, firing rates, and how these shift to a conserved meta plastic state during task acquisition. **(B)** Diagram illustrating causal versus acausal action potential generation across the corticostriatal circuit. **(C)** Diagram showing spike timing dependent plasticity driven by causal action potential generation across the corticostriatal circuit. **(D)** Diagram showing the temporal parameters behind spike timing dependent plasticity. **(E)** A diagram showing how the changes to E/I tone and firing rate support LTP induction during a brief window of plasticity for the NH and HL group. **(F)** A diagram showing the emergence of the neural response to the 4 Hz Go and 12 Hz No-Go stimulus in the striatum in the context of the default presence of the stimulus response in auditory cortex.

In this study we used an amplitude modulated auditory discrimination task, *in vivo* electrophysiological recordings, and *in vitro* whole cell recordings from auditory striatum to reveal neurophysiological differences during learning between normal hearing animals and those that had transient developmental hearing loss. Previous work had shown that the animals with transient hearing loss had significant differences in their baseline physiology (Mowery et al., 2017); however, there were no learning or performative differences between the groups (Paraouty and Mowery, 2021). This study aimed to reveal how the neurophysiological differences were compensated for at the population (*in vivo*) and cellular level (*in vitro*) to permit learning. The *in vivo* recordings revealed that over several days of training, neural activity associated with the nose poke, unmodulated sound, and response to reward was significantly reduced. At the same time, the neural response to the conditioning stimuli increased and phase locking emerged in both groups. As learning continued the decreases in neural activity related to non-conditioning stimuli returned to baseline levels and phase locking became more pronounced. The *in vitro* experiments demonstrated that the initial increase in phase locking to the auditory stimuli occurred contemporaneous to significant shifts in synaptic and intrinsic membrane physiology and increases to the probability of theta burst induced long-term potentiation (Figure 7). In general, the shifts in cellular and synaptic properties make the cells more likely to generate an action potential from glutamatergic inputs (Figure 7A). This configuration would favor causal synaptic events that lead to action potential generation (Figure 7B). As the regions investigated in this study are heavily innervated by cortical and thalamic glutamatergic inputs, the auditory stimulus would be an effective driver of forms of plasticity such as spike timing dependent plasticity (Figure 7C) especially when the medium spiny neurons begin to entrain to these inputs in a causal direction that underlies LTP versus LTD of the synapse (Figure 7D). This would be enhanced by contingency based (onset of NoGo trials) changes to dopamine release that support MSN depolarization, burst firing, and LTP (e.g., Wise and Jordan, 2021). Our results show that MSNs that shift to the cellular and synaptic plasticity state we refer to as “learning mode” are significantly more likely to undergo LTP after theta burst stimulation (Figure 7E). This “learning mode” likely supports the strengthening of the corticostriatal synapses and auditory neurons that are entrained to auditory stimuli in the default state (Figure 7F); thereby allowing them to become similarly entrained in what we observe as phase locking. The presence of a short temporal window for the “learning mode” of just a few days limits inadvertent association to non-conditioning stimuli and allows fidelity in learned associations once the window has closed.

Finally, it is important to note that the design of this study did not allow for distinction between the direct and indirect pathway, the differentiation of data into cell type specific analysis (D1 vs. D2), or the collection of FS interneurons. Recent studies using cre dependent mice that allow for differentiation between D1 and D2 receptors suggest that these two pathways are essential for auditory decision making in the tail of the striatum. In Cui et al. (2025) they found that unilateral activation of the direct or indirect pathway in the tail of the striatum biased decision making towards the opposite spout in an auditory head fixed licking task. Alternatively, inactivation of either pathway preferentially biased choice to that side; especially for the indirect pathway during the decision-making time window. Finally, the fast-spiking interneurons played a specific role, where disinhibition of both pathways biased decision making towards the opposite spout; especially in harder tasks. In Druart et al. (2025) they used cre dependent mice to differentiate the contribution of direct and indirect pathway activation by auditory inputs to the tail of the striatum. They found that direct pathway activation by either cortical or thalamic auditory inputs was more robust and temporally more aligned with the auditory stimulus. Local inhibition through stronger activation onto D2 MSNs lowered activation and produced a delay of activation of the indirect pathway to produce this effect. This result underscores the prevalence of the D1 MSN and direct pathway activation for auditory pathway plasticity. In context of the current findings reported here, further study should be carried out to reveal the contribution of the direct and indirect pathway, D1/D2 MSNs, and fast spiking interneuron towards the emergence of the auditory stimulus during learning, as well as, the role that cell type specific MSNs play in the brief window of plasticity associated with learning, LTP induction, and task acquisition.

### The suppression of non-associative neural activity (noise) allows for the emergence of neural phase locking to the associative conditioning signal

The way in which auditory cortex neurons follow the temporal envelope and temporal fine structure of amplitude and frequency modulated sounds is very similar among rodents, non-human primates, and humans (Bieser and Müller-Preuss, 1996; Hoglen et al., 2018). The Mongolian gerbil has exquisite auditory processing ability and can follow complex spectral and temporal envelopes and fine temporal structures (Penikis and Sanes, 2023). The neural processing of temporal sound cues emerge early in development through experience dependent interactions with the auditory environment (Zhang et al., 2001, 2002) and both frequency and amplitude modulation are coded in the active (attention modulated) and passively listening cortex (Niwa et al., 2015; Paltoglou et al., 2011). Cortex is classically considered the end of peripheral perceptual processing leading to the next question of how this encoded information is transformed into decision-making neural activity at a behavioral level. Corticostriatal entrainment of the medium spiny neurons in auditory cortex recipient striatum is a parsimonious explanation (Sameiro-Barbosa and Geiser, 2016). Across many different types of tasks, the temporal parameters of auditory conditioning stimuli are found to reliably activate auditory regions of the putamen in humans (Chen et al., 2008; Grahn and Rowe, 2009; Teki et al., 2011; Riecker et al., 2003; Grahn and Brett, 2007) and animals (Apicella et al., 1991a, 1991b; Winer, 2005; Arnauld et al., 1996; LeDoux et al., 1991; Bordi and LeDoux, 1992; Bordi et al., 1993; Hikosaka et al., 1989; McGeorge and Faull, 1989; Zhong et al., 2014; Reig and Silberberg, 2014; Chen et al., 2019; Guo et al., 2018, 2019; Nardoci et al., 2022; Ponvert and Jaramillo, 2019; Li et al., 2021). Through this cortical entrainment, auditory conditioning cues would produce medium spiny neuron phase locking to behaviorally relevant auditory drive from the cortex.

The recordings that we made in auditory striatum of adult animals improve upon this concept in the following ways. First, while there is a general innate response to sound onset early in training, the temporal information about the stimulus is largely absent. This might be because prior to the onset of NoGo trials, the Go auditory stimulus does not hold any contingency value. Changes in contingency, such as the addition of a negative valence component to the syndetic chain of behavior when the NoGo trial is introduced, allow the two auditory stimuli to now evoke plasticity along the circuit in order to reinstate the positive valence associated with food reward. For the two stimuli one salient feature is the difference in acoustic envelope parameters such as ramp speed, offset, and onset. Entrainment of neural activity to these robust features likely facilitate the neural discrimination between the two stimuli. For example, neural activity centered around the peak of the amplitude will allow three bursts of 12 Hz modulated action potentials to occur during a single cycle of 4 Hz modulation (250 ms). The difference in bursting activity could create a rate code that helps distinguish the two stimuli. Second, a new behavior quickly emerges where the animal stops or slows to listen to the auditory stimulus. In go training the animal learns to nose poke, which is immediately followed by movement to the trough to collect a food reward. As the striatum is ultimately a motor center this pause reduces the overall movement related activation of the medium spiny cells allowing the excitatory drive from the cortex to be a greater potentiation source. This effectively increases the signal to noise ratio of incoming glutamatergic input from auditory cortex. This leads to a second novel behavior chained to the pause that is directly related to the novel decision making that emerges in the task. Once the animal begins to learn to discriminate the two stimuli a repoke behavior is rapidly conditioned during the NoGo trial. Thirdly, once the signal emerges from the movement related background noise stimulus, phase locking by the medium spiny neurons gains more temporal fidelity to the corresponding cortical entrainment. This allows the animal to master the task by optimizing its ability to discriminate between the two stimuli, thus increasing the overall success rate to near perfection (*d*′ above 2.5). Finally, this initial learning provides a sort of behavioral scaffolding that allows more complex (harder) stimulus contingencies to be conditioned. Thus, it takes longer to initially train naïve animals on a discrimination task if the stimuli are too similar, but these same harder stimuli can be learned rapidly if the associative foundation is already present (Caras and Sanes, 2017).

This provides an exemplar model framework for the way in which abilities such as language acquisition build through foundational layers of experience dependent associative learning during development. It also improves our understanding of how missing sensory or behavioral experience during critical/sensitive periods of plasticity (such as language acquisition) can reduce, impair, or prevent learning/ability. Here it is important to note that permanent conductive hearing loss (malleus removal) does impair the acquisition of amplitude modulated/frequency modulated discrimination (Buran et al., 2014; Rosen et al., 2012; von Trapp et al., 2017; Yao and Sanes, 2018); however, our animal model of transient hearing loss, induces juvenile behavioral impairments, but allows a recovery of auditory perceptual processing to normal levels by adulthood (Anbuhl et al., 2022; Caras and Sanes, 2015). Future work will investigate how permanent perceptual impairments (e.g., noise induced hearing loss) delay learning through the corticostriatal circuit mechanisms discovered in this study and should provide more insight into therapeutic approaches across many perceptual-cognitive disorders.

### A brief window of plasticity allows cortical entrainment to potentiate corticostriatal pathways during associative learning

Activity dependent synaptic plasticity that produces long term potentiation is the classical model of learning, memory, and development discussed throughout all fields of neuroscience (Abbott and Nelson, 2000). Here the Hebbian concepts of fire together/wire together and spike timing dependent plasticity guide development and allow circuit remodeling later in life through long term potentiation and depression of active synapses. Ideally, circuits that involve a feedforward excitatory input and a labile recipient provide physiological opportunities for this type of plasticity. The corticostriatal circuit is an ideal model system to carry out investigations into this phenomenon, and many decades of work have demonstrated both LTP and LTD between layer 5 cortical neurons and their medium spiny neuron recipients in the striatum (Spencer and Murphy, 2000; Akopian et al., 2000; Calabresi et al., 1992a, 1992b; Charpier and Deniau, 1997; Fino et al., 2010). In this study we used this circuit as a model system to investigate how developmental hearing loss alters the synaptic plasticity associated with learning. We used a theta burst protocol that permits the study of long-term potentiation and depression in the adult corticostriatal brain slice (Hawes et al., 2013) to ask how the probability of LTP changes during associative task learning.

In our previous study we demonstrated how GABAergic inhibition is altered in normal hearing and hearing loss animals during task acquisition (Paraouty and Mowery, 2021). The results suggested a key role of GABAergic disinhibition in learning. Here we replicated those findings and extended them out to 10 days (T9–T10) showing that inhibitory synaptic plasticity is reversibly altered only during a very brief window of learning. The change to inhibitory tone is thought to support LTP induction through disinhibition, which increases excitability and allows maintenance of active synapses in adult circuits (Stelzer et al., 1994; Kotak et al., 2013; Williams and Holtmaat, 2019). Under normal stimulation conditions (adult E/I tones) feedforward activation often leads to long term depression of synapses. Pharmacological, optogenetic, and chemogenetic manipulation of inhibition (typically suppression) coupled with high frequency, paired, or theta burst stimulation reliably induces LTP across many circuits (Skiteva et al., 2018; Kotak et al., 2013; Ito et al., 2020; Ormond and Woodin, 2009, 2011; Rodrigues et al., 2021). This is related to increased postsynaptic excitability through AMPA receptor release by the GABAA receptor suppression and both presynaptic GABAB auto receptor activation (that reduces GABA synaptic release) and postsynaptic NMDA release by GABAB receptor activity reduction (Davies and Collingridge, 1996; Mott and Lewis, 1991, 1992). Here NMDA receptor activation is the key component to the induction of LTP over LTD (Aroniadou and Teyler, 1991; Dozmorov et al., 2006; Lüscher and Malenka, 2012; Wang et al., 2002; Murphy et al., 1997). In these experimental conditions pharmacological receptor manipulation is required to nudge excitatory and inhibitory receptors into desired states that promote long term potentiation.

The current findings from our study are confirmatory of this previous research in an *in vivo* model system. Thus, we have confirmed that the natural plasticity mechanisms that drive LTP and learning *in vivo* are the same as previously described in the laboratory experiment. First, the reduction in inhibitory tone allows feedforward glutamatergic output to exert more potentiation action. Each stimulus presentation has an increased probability of inducing an action potential in the medium spiny neuron. Second, the elevated synaptic excitability increases the probability of membrane depolarization, action potential generation, and NMDA receptor activation via magnesium block removal (Calabresi et al., 1992b). This increases the probability that repeated exposure to the stimulus will lead to potentiation between the corticostriatal synapses, and we confirm this with theta burst induced increases in LTP expression in our auditory corticostriatal slice preparations. *In vivo*, the brief window of plasticity increases the probability that stimulus response associations are potentiated, and we see this as (1) the emergence of the neuronal population response (stimulus phase locking) to the two auditory stimuli that allows discrimination and (2) behavioral reinforcement (reward) through the establishment of the re-poke correct rejection syndactic chain that leads to a decrease in false alarms to near zero as animals master the task. The default state for the HL animals was counter intuitive to this notion as they begin training in a cellular state that is already highly excitable. It is important to note that medium spiny intrinsic physiology is significantly suppressed in the default state of HL animals reducing evoked firing rates, and likely to prevent rampant aberrant potentiation to inadvertent peripheral stimulation. This intrinsic suppression briefly returns to a normal state during behavioral acquisition. This serves to underscore the precise temporal nature of spike timing dependent plasticity and LTP (Dan and Poo, 2004), wherein causal feedforward activity that leads to an action potential has a definitive temporal window that relies on a “learning mode” state of cellular and synaptic excitability that could be a universal meta plasticity associated with associative conditioning.

### A role for dopamine to open and close the brief window of learning plasticity

This brief window of plasticity could allow synaptic remodeling of existing circuit pathways to establish robust and novel stimulus response associations. A major factor for this entire theoretical framework will involve tonic and phasic release of dopamine and other neuromodulators that contribute to LTP and learning (Cui et al., 2015; Reynolds et al., 2022; Hawes et al., 2013). Importantly, recent work in the posterior tail of the striatum has highlighted the role of dopamine in reinforcement learning. First, infusion of a dopamine antagonist (D1/D5) into the tail of the striatum reduced learning while an agonist increased the learning of this same auditory task in gerbils (Paraouty et al., 2021). Measuring the compartmentalized release of dopamine in the posterior tail of the striatum shows that there is a significant concentration of release in the lateral auditory regions where we implant our electrodes and carry out our *in vitro* recordings (Riley et al., 2024). Furthermore, dopamine release in the tail of the striatum has a significant potentiating effect on MSNs (D1) especially for novel sensory stimuli (Tsutsui-Kimura et al., 2025). This would be a factor during the introduction of the NoGo auditory stimulus and based on these results could be a major driver of dopamine release. This could be the physiological event that signals the opening of the brief temporal window associated with task acquisition. To that end, the amount of dopamine release in the tail of the striatum is correlated with the intensity of a novel stimulus (Siciliano et al., 2018). This could explain the increase in the neural response to the conditioning auditory stimuli over time allowing the phase locking to first emerge in later cycles of stimulus delivery. Aside from changes to E/I synaptic tone and cellular intrinsic excitability the effect of increased dopamine during this brief window of plasticity could facilitate potentiation of the medium spiny corticostriatal and thalamostriatal synapses during auditory stimulus activation. For example, higher concentration levels of dopamine are associated with the induction of LTP in striatal fast spiking GABAergic interneurons and medium spiny neurons (Calabresi et al., 1997; Centonze et al., 1999, 2001; Thivierge et al., 2007), which we might expect based on the way the tail of the striatum responds to novel/intense stimuli. To that end, the presence of increased dopamine during this brief window would work synergistically with the “learning mode” plasticity state we describe here. It would facilitate spike timing dependent forms of LTP (via burst firing) that lead to the establishment of corticostriatal circuits that respond preferentially to the conditioned stimulus (Pawlak and Kerr, 2008). As the novelty of the conditioning stimulus is reduced and the reward contingency is regained by the emergence of the correct rejection behavior, a reduction in DA that opened the window might also close it. To explore this, further work could manipulate and measure levels of dopamine during a task like this to establish how it interacts with the brief window of learning plasticity that we have identified in this study.

## Data Availability

The raw data supporting the conclusions of this article will be made available by the authors, without undue reservation.
